# Where is the game? Wild meat products authentication in South Africa: a case study

**DOI:** 10.1186/2041-2223-4-6

**Published:** 2013-03-01

**Authors:** Maria Eugenia D’Amato, Evguenia Alechine, Kevin Wesley Cloete, Sean Davison, Daniel Corach

**Affiliations:** 1 Biotechnology Department, Forensic DNA Lab, University of the Western Cape, Modderdam Road, Bellville, 7535, South Africa; 2 Servicio de Huellas Digitales Genéticas, School of Pharmacy and Biochemistry, University of Buenos Aires, Junín 956, Buenos Aires, 1113, Argentina

**Keywords:** Biltong, conservation, Cytb, DNA barcoding, Food forensics, Meat, Species identification

## Abstract

**Background:**

Wild animals’ meat is extensively consumed in South Africa, being obtained either from ranching, farming or hunting. To test the authenticity of the commercial labels of meat products in the local market, we obtained DNA sequence information from 146 samples (14 beef and 132 game labels) for barcoding cytochrome c oxidase subunit I and partial cytochrome b and mitochondrial fragments. The reliability of species assignments were evaluated using BLAST searches in GenBank, maximum likelihood phylogenetic analysis and the character-based method implemented in BLOG. The Kimura-2-parameter intra- and interspecific variation was evaluated for all matched species.

**Results:**

The combined application of similarity, phylogenetic and character-based methods proved successful in species identification. Game meat samples showed 76.5% substitution, no beef samples were substituted. The substitutions showed a variety of domestic species (cattle, horse, pig, lamb), common game species in the market (kudu, gemsbok, ostrich, impala, springbok), uncommon species in the market (giraffe, waterbuck, bushbuck, duiker, mountain zebra) and extra-continental species (kangaroo). The mountain zebra *Equus zebra* is an International Union for Conservation of Nature (IUCN) red listed species. We also detected *Damaliscus pygargus*, which is composed of two subspecies with one listed by IUCN as ‘near threatened’; however, these mitochondrial fragments were insufficient to distinguish between the subspecies. The genetic distance between African ungulate species often overlaps with within-species distance in cases of recent speciation events, and strong phylogeographic structure determines within-species distances that are similar to the commonly accepted distances between species.

**Conclusions:**

The reliability of commercial labeling of game meat in South Africa is very poor. The extensive substitution of wild game has important implications for conservation and commerce, and for the consumers making decisions on the basis of health, religious beliefs or personal choices.

Distance would be a poor indicator for identification of African ungulates species. The efficiency of the character-based method is reliant upon availability of large reference data. The current higher availability of cytochrome b data would make this the marker of choice for African ungulates. The encountered problems of incomplete or erroneous information in databases are discussed.

## Background

The consumption of game meat is popular in southern Africa, especially in dry form. The consumption of wild ungulates - loosely called ‘game’ - and ostrich is viewed as a healthy alternative to beef, because of their low content of fat and cholesterol [1] and for the natural origin of game products, devoid of antibiotics, anabolic steroids, hormones and other additives, in an increasingly health-aware market. Game meat is consumed mostly dry in southern Africa. Traditional preparations have been locally consumed since colony times: ‘biltong’ consists of strips of meat seasoned with vinegar and spices and dried with hot air, whereas ‘droë wors’ are simply hot-air dried sausages for long-term storage. The market trend in favor of game meat is reflected in the increase in ranching from 600,000 head of game in 1964 to 18.6 million in 2007, with a result that 80% of game animals are being kept on private land [2]. Currently, 20.5 million ha of marginal agricultural land are owned by over 10,000 commercial wildlife ranching farms containing 2.5 million heads of game. This commercial activity is predominantly driven by the dry meat demand [2,3]. Hunting is also an important source of wild meat for the specific purpose of biltong production [4]. A conservative estimation of the magnitude of this activity indicates that over 1 million animals are hunted yearly (Peet van der Merwe, personal communication), with a contribution to gross domestic product that exceeds ZAR 6 billion (USD 750 million) [5]. The preferential targets are springbok (*Antidorcas marsupialis*), impala (*Aepyceros melampus*), blesbok (*Damaliscus pygargus phillipsi*) and kudu (*Tragelaphus strepsiceros*). Intensive farming is reserved for ostrich (*Struthio camelus*) production.

Ranched and hunted game in South Africa and Namibia is distributed in the form of dressed carcasses to supermarkets and butcheries by wholesalers or hunters. According to the South African Meat Safety Act 40 of 2002 [6], meat from wild animals sold for human consumption must be accompanied by a permit, and the meat must be processed by an accredited abattoir and approved upon regulated inspection. However, these regulations do not apply to biltong hunters [7], who most frequently butcher the hunt themselves [8].

Biltong is manufactured both industrially and in small-scale family businesses, resulting in a mixed market of branded and unbranded products. In South Africa, the game industry is a free market enterprise devoid of central marketing structure [3]. The labeling of game meat and biltong relies largely - or solely - on wholesalers and manufacturers. The delivery of head- and skin-off dressed carcasses and the general lack of regulations increase the chances of species mislabeling and product substitution or fraud. It is surprising that the list of most frequently hunted game shows over 20 species [8] whereas only seven can be found in the local market: springbok, kudu, gemsbok (*Oryx gazella*), impala, eland (*Tragelaphus oryx*), wildebeest (*Connochaetes* species) and ostrich.

Of no less concern is the possibility of intentional delivery of endangered species in the meat market. In South Africa, the Convention on International Trade in Endangered Species of Wild Fauna and Flora (CITES) [9] listed endangered ungulate species are the bontebok *Damaliscus pygargus pygargus* (synonym *D*. *dorcas dorcas*, *D*. *p*. *dorcas*) (CITES Appendix II), the cape mountain zebra *Equus zebra zebra* (CITES Appendix I), the southern white rhinoceros *Ceratotherium simum simum* (CITES Appendix I), the black rhinoceros *Diceros bicornis* (CITES Appendix I) and the African elephant *Loxodonta africana* (CITES Appendix II). The International Union for Conservation of Nature (IUCN) Red List of Threatened Species 2012 [10] catalogued these species as ‘vulnerable’ (*E. zebra*, *L. africana*), ‘critically endangered’ (*Diceros bicornis*), ‘near threatened’ (*Ceratotherium simum simum*) and ‘of least concern’ (*D*. *p*. *pygargus*).

The identity of meat and other wildlife products is of common interest for both food science and protection of biodiversity. The most frequently applied techniques involve the analysis of markers such as mitochondrial DNA (mtDNA) fragments of cytochrome b (cytb), D-loop, cytochrome c oxidase subunit I (COI), 12S rRNA and 16S rRNA coding regions, and STRs with a variety of typing techniques reviewed in [11-14].

The early availability of universal primers for cytb [15,16] fuelled studies of molecular evolution and made a large number of sequences available. The most studied cytb fragment is 358 bp long, and its relatively high level of intraspecific and interspecific variation made it attractive for phylogenetic and phylographic studies. The widespread application of COI in conservation and evolution is more recent, resulting from the establishment of the Barcode of Life Data Systems (BOLD) database [17,18]. This situation determines a higher availability of information for cytb. The cytb:COI ratio for Cetartiodactyla (even-toed ungulates) and Perissodactyla (odd-toed ungulates) taxa represented in GenBank (accessed in July 2012) is 2.5 and 4.5 respectively.

This COI underrepresentation bias will likely be reversed with the growth of the BOLD database. BOLD banks DNA sequence information of a 648 bp fragment of COI, coined ‘barcode’, along with other valuable biological data such as voucher number and institution of origin. The high quality control of DNA sequence information to identify a species is a clear advantage over GenBank, in which the deposition of false sequences has been reported [19-21].

The suitability of cytb versus COI in animal forensics has long been debated. A study by Tobe *et al*. [22] provided a guideline for the reliability of species identification using cytb and COI. Compared with COI, cytb demonstrated a higher phylogenetic signal and a higher power to correctly identify species, therefore making cytb a more attractive marker for forensic applications. Tobe *et al.* used whole mitochondrial genome sequences of *Canis*, *Homo* and *Bos* and 236 cytb and COI mammal sequences, and estimated the within-species variation to be lower than a Kimura-2-parameter (K2P) distance (×100) of 1.5 for both mtDNA fragments, whereas the differences between species had a K2P (×100) value higher than 2.5. Using the COI barcoding region, Hebert *et al*. [17] suggested a 3% threshold of sequence divergence for within-species variation. Subsequently, it was suggested a 10-fold rule for within-species versus between-species K2P genetic distance [23].

Most DNA sequence information available for African ungulates comes from phylogenetic and phylogeographic studies using the cytb or D-loop region. The application of cytb and COI to conservation and food studies is very scarce in Africa: COI barcoding sequences have been made available for central and western African mammalian species [24-27]. To date, the sole study of species authenticity in the South African commercial market was conducted on fish species using 16S rDNA and 84% substitution was evidenced [28].

Here, we present a case study of species identification in wild meat food products in South Africa. The identifications were performed by means of comparative DNA sequence information using the mtDNA gene fragments cytb and COI. We applied three different methods: a similarity method implemented in the National Center for Biotechnology Information GenBank database [29]; a phylogenetic method to identify monophyletic clusters; and a character-based logic mining method that infers diagnostic characters from reference sequences for further classification [30]. The significance of matches in GenBank was further evaluated by means of phylogenetic reconstruction and by evaluation of the extent of known variation within and between species. By following this approach we intend to overcome the possibility of identification uncertainty arising from incomplete taxa availability; incomplete or lack of within-species genetic information in databases; and evolutionary events such as incomplete lineage sorting, secondary contact and consequent hybridization, cryptic speciation, and other populations processes. This is the first study of this nature for game food commercial products conducted in South Africa.

## Methods

We applied basic laboratory practices according to Budowle *et al*. [31] and followed the recommendations of the International Society for Forensic Genetics for non-human DNA typing [32].

### Samples

A total of 146 samples were collected from wholesalers, supermarkets and outlets. Labels indicated the collected products were beef (*Bos taurus* N = 14), generic ‘game’ (N = 6), springbok (N = 33), gemsbok (N = 14), impala (N = 4), blesbok (N = 2), kudu (N = 38), eland (N = 8), blue wildebeest (*Connochaetes taurinus*) (N = 1), ostrich (N = 23), zebra (either cape mountain or Burchell’s zebra *E*. *quagga burchellii*^a^) (N = 1) and warthog (*Phacochoerus africanus*) (N = 2). All beef samples consisted of biltong, whereas game samples were obtained in the form of biltong (N = 94), droë wors (dry sausages) (N = 30), fresh and minced meat (N = 10), carpaccio (N = 6), fresh sausages (N = 2) and smoked meat (N = 4).

Voucher specimens with known classification and relevant collection information (photo and global positioning system co-ordinate) were collected. Reference samples for the following species was obtained either from the National Zoological Gardens of South Africa, South African National Parks and the Quagga Project Association or with the assistance of various South African conservation agencies: cape mountain zebra, (N = 6), Burchell’s zebra (N = 2), bontebok (N = 5), blesbok (N = 4), black wildebeest (*Connochaetes gnou*, N = 2), blue wildebeest (N = 3), springbok (N = 3), nyala (*Tragelaphus angasii*, N = 1), eland (N = 1), gemsbok (N = 1). The origin and species of these samples are known as all zoos participate in animal record-keeping databases and the International Species Information System and confirm speciation according to IUCN guidelines.

Single DNA extractions and analyses were performed for biltong, carpaccio, fresh meat and smoked meat whereas, for fresh and dry sausages, DNA sequence information was obtained from at least two small samples from different dissected fractions of approximately ≤1 mm^2^ (fat and meat). DNA was extracted following a salting-out method [33] modified by increasing the proteinase K concentration to 0.1 mg/ml in a lysis volume of 500 μL. DNA was resuspended in 50 μL bidistilled water and quantified using a Nanodrop ND-2000 spectrophotometer (Thermo Fisher Scientific, Wilmington, USA).

### PCR and sequencing

DNA polymorphisms were analyzed for cytb and COI. We used the universal vertebrate cytb primers L14816 5^′^-CCATCCAACATCTCAGCATGATGAAA-3^′^ and H15173 5^′^-CCCCTCGAATGATATTTGTCCTCA-3^′^[34], positioned between sites 14583 and 14941 in the *B. taurus* mtDNA reference sequence V00654 [35]. COI barcoding region primers [36] were redesigned to overcome difficulties found during amplification. The primers L5701 5^′^-CTGAYTATTYTCAACYAACCAYAAAGA-3^′^ and H6418 5^′^-ATAKACTTCRGGGTGTCCRAAGAATCA-3^′^ were designated according to their position and amplicon size using the *B. taurus* mtDNA reference sequence V00654 [35]. *Macropus*-specific COI primers were designed for samples with mixtures of two or more species after *Macropus* species were identified from the same samples with cytb sequence data: MACR-COI-F 5^′^-TAGGAACTGCCTTAAGTCTGCTC-3^′^ and MACR-COI-R 5^′^-TGAAAGGAGAAGGAGGACTGCTG-3^′^. Amplifications were performed in a Verity Thermal Cycler (Applied Biosystems, Singapore) in a final volume of 25 μL containing 25 ng DNA, 2 mM deoxyribonucleotide triphosphates, 0.4 mM primers, 1 U Super Therm Taq polymerase (JMR Holdings, Kent, UK). PCR cycling conditions were 94°C for 3 min followed by 35 cycles of 94°C for 30 s, 45°C (cytb) or 50°C (COI) for 30 s, and 72°C for 45 s with a final extension time of 72°C for 10 min. PCR products were checked on 2% agarose gels in 1× tris-borate-EDTA buffer, and sequencing reactions in both directions were either outsourced to Macrogen (Seoul, South Korea), or performed in our facilities in 10 μl reaction final volume with BigDye Terminator v 3.1 (Life Technologies, Foster City, CA, USA). Sequencing products were resolved in an ABI 3130XL (Applied Biosystems, Japan) at the Central Analytical Facility of Stellenbosch University and in our facilities.

### Species identification, validation and data analyses

Species DNA identification was initially assessed by BLAST [29] searches in GenBank [37] using the maximum similarity values from pairwise alignments. All available entries for the matched species and related species from the same genera and tribe were used for phylogenetic analysis and for evaluating the range of genetic variation within and between species. Entries from environmental samples (for example, food, mosquito bloodmeal) were excluded from our analyses. GenBank accession numbers of sequences used for all analyses are provided in Table S1 in Additional file 1.

DNA sequence alignments were performed with Clustal W implemented in BioEdit Sequence Alignment Editor 7.0.9.0 [38]. Mean K2P [39] genetic distance was calculated for unique sequences within species, and net between-groups mean K2P distance was calculated between species. Standard errors were calculated with 1,000 bootstrap replicates. Sister species were identified either from whole mitochondrial genomes in the phylogenetic analysis of Hassanin *et al*. [40], or from our phylogenetic analysis.

Maximum likelihood (ML) phylogenetic trees were constructed using the K2P substitution model with uniform rates among sites, treating missing data using the partial-deletion option, and applying the Nearest Neighbor Interchange heuristic method. Branch support was evaluated with 1,000 bootstraps as implemented in MEGA v. 5.0 [41].

The character-based method implemented in BLOG v. 2.0 [42] was applied to the identification of ‘classes’ (species) in each alignment. This method extracts diagnostic characters from reference sequences for the classes (species) used for ‘training’ and infers diagnostic formulae for each class using a learning algorithm. These formulae are applied to classify the test sequences. The padding parameter was set to 1 for the uneven length of some reference sequences; all other parameters were applied at default values. BLOG was shown to perform better than similarity and neighbor-joining, parsimony or Bayesian phylogenetic methods [42].

Identifications were scored as ‘correct’ when cytb and COI DNA sequence information obtained from multiple dissections indicated the same species as the commercial label, and ‘false’ when either both or one DNA fragment was non-coincidental with the species indicated by the product label.

## Results and discussion

### Market substitution statistics

A total of 151 COI [GenBank:JX567005-JX567156] and 152 cytb [GenBank:JX567157-JX157309] DNA sequences were obtained from the 146 collected food samples, and 29 COI [GenBank:JX436976-JX436996; JX566994-JX567001] and seven cytb [GenBank:JX436997-JX437000; JX567002-JX567004] DNA sequences from known reference samples. Detailed information of the food samples, GenBank accession numbers, and identification results are provided in Table S2 in Additional file 2.

The cytb and COI analysis of 146 commercial red meat samples indicated that 101 were false (69.18%). Excluding the samples collected as ‘beef’, which were all correctly labeled, the strict substitution of wild meat then becomes 76.5% (Table 1). Of all substitutions, 49 samples indicated domestic or alien species: thirty-five beef, one horse (*E*. *caballus*), six kangaroo (*Macropus giganteus*, *M*. *rufus*, and *M*. *robustus* and an unidentified *Macropus* species), one pork (*Sus scrofa*) and seven lamb (*Ovis aries*). All other substitutions indicated other African wild species: giraffe (*Giraffa camelopardalis*), nyala, kudu, bushbuck (*T. scriptus*), blesbok, blue wildebeest, hartebeest (*Alcelaphus buselaphus*), waterbuck (*Kobus ellipsiprymnus*), gemsbok, zebra (*E. zebra*) and common duiker (*Sylvicapra grimmia*).

**Table 1:** Extent of substitutions summarized by product type

**Sample type**	**N**	**False**	**Percentage substitution**
Biltong	94	65	69.15
Carpaccio	6	2	33.33
Dry sausage	29	25	86.21
Fresh sausage	2	1	50.00
Mince and fresh	10	5	50.00
Smoked	5	3	60.00
**Total**	**146**	**101**	**69.18**

N= number of samples.

The seemingly relaxed nature of the meat trade may mean that not all substitutions of native game with other game were intentional. In addition, most shops display biltong in open baskets, and the misplacing of labels cannot be discarded. Nevertheless, the substitutions with domestic animals, kangaroo and game meat normally absent from the market (for example, giraffe) cannot be attributed to human error but must be regarded as intentional. Given the richness of antelopes and other bovids in South Africa, the limited number of species in the market is surprising, likely due to a commercial simplification of choices for consumers with poor wildlife knowledge.

### Validation of species identifications

A simple similarity value is meaningless unless the range of variation within the class is known. We followed different methods to estimate the reliability of our assignments. The assignments were considered correct when the BLAST similarity value score was 100%, and the query sequence belonged to monophyletic clusters with high bootstrap support (Figure S1a-r in Additional file 3). All cytb and COI sequences obtained in this study showed identities higher than 97% with existing sequences in the GenBank database (Table S2 in Additional file 2).

**Figure 1:**
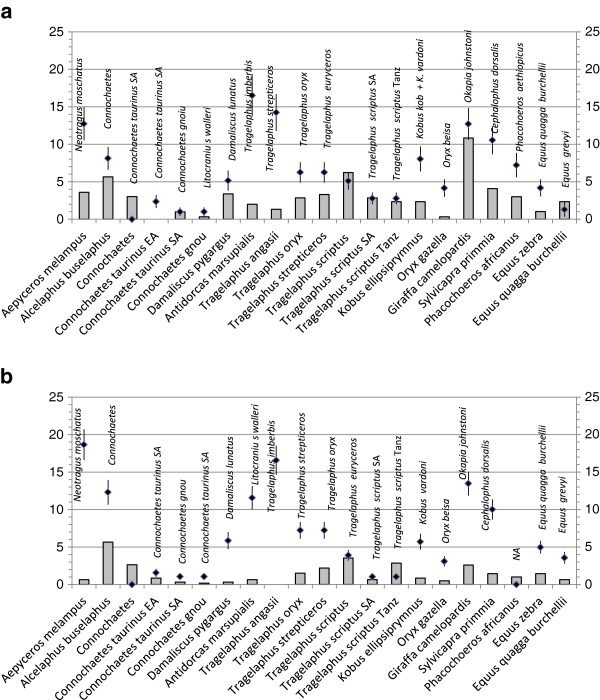
**Kimura 2-parameter distances (×100) observed within and between species. (a)** Maximum observed cytochrome b Kimura 2-parameter (K2P) distance within species are shown in histograms, bars indicate the net between-species K2P divergence from the phylogenetically closest related species. **(b)** Maximum observed cytochrome c oxidase subunit I K2P distance within species are shown in histograms, bars indicate the net between-species K2P divergence from the phylogenetically closest related species. NA: Not available, SA: South Africa, Tan: Tanzania.

The intraspecific and interspecific range of genetic variation was plotted for all identified native African groups except ostrich (Figure 1a,b). Genetic variation within and between species that do not conform to published recommendations [17,21-23] for species delimitation are discussed in the following section for each case.

Phylogenetic trees were not shown when scarce information was available (Giraffidae, Suidae and Macropodidae COI) or for trees with similar information to that recovered by cytb (Bovini, Caprini). The BLOG classes that identified query and test DNA fragments correctly with 100% efficiency are shown in the phylogenetic tree figures. Incorrect assignments occurred when data contained insufficient information or the availability of references to test sequences did not reach the required 4:1 proportion.

Below we present the evaluation of species identifications for each group, and discuss the results in view of the known variation and evolutionary history of each group.

#### Bovidae: Antilopinae: Aepycerotini

This tribe is represented by one monotypic genus, represented by the impala *Aepyceros melampu*s, with two subspecies with disjunct distribution: *A*.*m*. *petersi* and *A*. *m*. *melampus*. It is a very common species in the grasslands and bushlands of eastern south-central and southeastern Africa. Two out of the four samples labeled ‘impala’ were correct, and was found as substitute for other game.

The genetic identification of this species would not pose any concern as it appears basal to Antilopini [40], with large sequence divergence from its relatives [40]. The COI phylogenetic tree is shown in Figure S1a in Additional file 3.

Both cytb and D-loop detect continental and regional (South Africa) phylogeographic structure respectively [43,44]. The COI phylogenetic tree reflects a similar continental pattern to that of cytb [43]. However, the frequent translocations of this species in South Africa (see below) would hamper the inference of local geographic origin of samples.

#### Bovidae: Antilopinae: Antilopini

This tribe is represented by gazelles, which inhabit open semiarid environments in Africa and Eurasia. Springbok is one of the most abundant antelopes in southern Africa. It was found mostly substituted (76%) with domestic, foreign or other game species and as a substitute for ostrich (Table 2).

**Table 2:** Substitutions found for meat products with cytb and COI DNA sequence information

					**Substituted samples**			
**Commercial label**	**Species**	**N**	**Correct**	**False**	**Substituted with beef**	**Mixture of species**	**Other species found in non-mixed samples**	**Species found in mixed samples (sausages)**
Beef	*Bos taurus*	14	14	0	NA	1	NA	
Blesbok	*Damaliscus pygargus*	2	1	1	0	0	Bushbuck	-
Blue wildebeest	*Connochaetes taurinus*	1	0	1	0	-	Nyala	-
Eland	*Tragelaphus oryx*	8	1	7	2	-	Gemsbok, kudu, blue wildebeest,	-
Game	Game	5	2	3	3	0	Gemsbok	-
Gemsbok	*Oryx gazella*	14	4	10	3	-	Kudu, blesbok or bontebok, blue wildebeest, impala	-
Impala	*Aepyceros melampus*	4	2	2	1	-	Bontebok or blesbok	-
Kudu	*Tragelaphus strepsiceros*	39	3	36	13	5	Hartebeest, gemsbok, waterbuck, eland, mountain zebra, giraffe, lamb, kangaroo	Kangaroo + beef, beef + lamb , eland + hartebeest
Ostrich	*Struthio camelus*	23	7	16	10	5	Springbok, kudu, black wildebeest, kangaroo	Ostrich + lamb, beef + pork, beef + lamb
Springbok	*Antidorcas marsupialis*	33	8	25	11	4	Gemsbok, kudu, eland, blue wildebeest, hartebeest, impala, common duiker, kangaroo, horse	Beef + waterbuck, beef + lamb, springbok + lamb
Warthog	*Phacochoerus africanus*	2	2	0	0	0	-	-
Zebra	*Equus burchelli*	1	1	0	0	0	-	-
**Total**		**146**	**45**	**101**	**43**	**15**		

The number of substituted products in the form of species mixtures is indicated in the column ‘**Mixture of species**’, and the nature of these mixtures are indicated under “**Species found in mixed samples (sausages)”**. The substitutions for biltong, fresh meat, carpaccio and smoked meat are summarized under ‘**Other species found in non-mixed samples”**’. “**Substituted with beef” is indicated for both mixed and non-mixed samples.** Domestic and foreign substitute species are indicated in bold. NA: not applicable.

The maximum sequence variation observed for this species was 2% for cytb and 0.6% for COI. For the scarce information available, these values should be taken with caution. The COI phylogenetic tree is shown in Figure S1a in Additional file 3.

#### Bovidae: Antilopinae: Alcephalini

Four genera compose this tribe of grazers, herd antelopes: *Alcelaphus*, *Beatragus Connochaetes* and *Damaliscus*; only *Beatragus* does not occur in southern Africa.

*Damaliscus pygargus* are distinctive white-faced antelopes endemic to South Africa, divided into subspecies by geographic range: bontebok in coastal fynbos and blesbok in the Highveld. The bontebok has recovered to a current number of approximately 3,500 animals with a consequent change of their IUCN status ‘vulnerable’ to ‘near threatened’ in 2008. One out of two collected samples of blesbok was correct, and it was found as a substitute for other game (Table 2).

The information contained in cytb and COI fragments did not allow for subspecies resolution with either the phylogenetic or the character-based methods (Figure S1b,c in Additional file 3), possibly because of incomplete lineage sorting or hybridization. D-loop was reported to distinguish these two forms [45]. The original discontinuous geographic distribution of these forms has been blurred by translocations to private land and other anthropogenic activities bringing them into secondary contact, resulting in hybridization. Therefore, for food validation purposes, the identification at the subspecies level may imply an unnecessary effort.

The hartebeest was found as a substitute for other game. Flagstad *et al*. [46] identified two major continental lineages with D-loop and cytb, which would allow for inference of geographic origin of samples at continental level. The cytb phylogenetic analysis shown in Figure S1b in Additional file 3 indicates the identity of our samples with *A*. *b caama*; incomplete taxa availability for COI forces our sequences to cluster with *A*. *b*. *lichtensteinii* (Figure S1c in Additional file 3). BLOG results show 100% efficiency assignment to *A. buselaphus* with cytb and one element remained not classified for a polymorphism in a ‘diagnostic’ site (Figure S1b in Additional file 3).

The black wildebeest is endemic to southern Africa, whereas the blue wildebeest occurs in eastern and southern Africa. They display morphological, behavioral and ecological differences. Wildebeest was found substituted by and substituting for other game species (Table 2). The low genetic variation observed for the genus falls within that observed for other within-species values (Figure 1a,b). These results are evidence of a very recent speciation event. In addition, the black wildebeest experienced extensive hunting and habitat reduction that resulted in a drastic reduction of its population size [40] and references therein]. Its IUCN status was ‘vulnerable’ until 1994, but since 2002 it is considered of ‘no concern’. Despite its recovery, the important reduction in population size is still reflected in the low level of genetic variation found in cytb and COI fragments.

Using D-loop, significant phylogeographic structure has been detected for the blue wildebeest, with clusters specific to eastern Africa and southern Africa [47]. Our phylogenetic analyses with cytb and COI showed a similar geographic dichotomy, with its southern form being more closely related to the black wildebeest. BLOG recovered complete assignments for both species with COI information and assignment to a single class containing all *Connochaetes* cytb sequences.

Hybridization between wildebeest species is known in South Africa as a result of anthropogenic translocations beyond their natural geographic range [48]. This phenomenon is reflected among our reference South African blue wildebeest samples, which cluster with black wildebeest (Figure S1c in Additional file 3). Therefore, for food identification purposes, wildebeest should be certified only at the level of genus in southern Africa.

#### Bovidae: Antilopinae: Caprini

Lamb was found in kudu biltong, in ostrich and springbok droë wors, and mixed in venison (springbok) mince. Phylogenetic trees with other caprini showed a monophyletic cluster with high bootstrap support for our samples (Figure S1d in Additional file 3).

#### Bovidae: Bovinae: Bovini

All samples labeled beef were correct. However beef was found present in 32% of samples labeled as some form of game.

The cytb and COI phylogenetic trees with other bovini showed a monophyletic cluster with high bootstrap support for our samples. The cytb tree is shown in Figure S1e in Additional file 3.

#### Bovidae: Bovinae: Tragelaphini

Tragelaphini is a tribe of spiral-horned, large antelopes. Kudu inhabits eastern and southern Africa; 92% of the kudu samples were substituted with domestic, foreign or other game species (Table 2).

Our cytb BLAST searches identified high similarity to entries [GenBank:L13794.1] and [GenBank:L13793.1], submitted as *Bubalus depressicornis*, an Indonesian buffalo species. The phylogenetic reconstruction of Figure S1f in Additional file 3 and BLOG assignments confirmed the identity of [GenBank:L13794.1] to kudu and [GenBank:L13793.1] to eland.

The maximum within-species variation was 3.293% for cytb and 2.183% for COI (Figure 1a,b), exceeding the average divergence within-species values indicated by Tobe *et al*. [22]. Phylogenetic trees show kudu sequences arranged into two clusters, corresponding to eastern and southern Africa. In turn, the southern African sequences are arranged in two clusters. Nersting and Arctander [43] showed continental phylogeographic structure with D-loop analysis. Unfortunately, no samples from South Africa were used in this study.

The eland is a very large antelope species, with males reaching 600 kg. It inhabits savannahs and grasslands of eastern and southern Africa. Only one out of the eight eland samples was correct. This species was substituted with beef and other game, and was utilized as a substitute of other game species (Table 2). Similarly to the results for kudu, eland sequences are arranged in two clusters corresponding to eastern and southern Africa (Figure S1f,g in Additional file 3). This structure was previously observed using D-loop sequence information [49].

The nyala is endemic to southeastern Africa where it inhabits forests and woodlands close to water. This species was found as a substitution for blue wildebeest. Very little information is available for this species, thus the values in Figure 1 should be taken with caution. A significant population structure was detected using microsatellites and D-loop [50], which could potentially be used to infer the geographic origin of samples.

The bushbuck is a browser species, widely distributed in forests and bushlands on the continent. This species was found as a substitute for blesbok. The differential clustering of the southern African and Cameroonian bushbuck (Figure S1f,g in Additional file 3) is due to the known paraphyly of this group [51]. Hassanin *et al*. [40] suggested they should be considered different species.

Figure S1a,b in Additional file 3 shows an inflated within-species variation because it is composed of two different taxonomic units. To account for this taxonomic problem, we plotted the within-species variation for *T*. *scriptus* from South Africa and Tanzania separately (Figure 1). Notwithstanding the overlapping within-between species genetic distance, the phylogenetic trees clearly distinguished them. BLOG recognized three classes with 100% efficiency for cytb data, but recognized one single bushbuck class with COI data.

#### Bovidae: Bovinae: Hippotragini

This tribe is composed of genera *Hippotragus* (saber and roan antelopes), *Addax* and *Oryx*. The four *Oryx* species inhabits semi-desert areas. The southern African oryx or gemsbok is found in South Africa and Namibia.

Most gemsbok samples were substituted with beef and other game, and it was also found as substitute for other game species (Table 2). A surprisingly low level of genetic variation seems to be harbored by this species (Figure 1), with one variable site for cytb and six for COI. The phylogenetic trees in Figure S1h,i in Additional file 3 show an odd clustering for JN869311 (*Oryx dammah*), which is likely an erroneous submission of *Addax nasomaculatus*. BLOG identifies *A*. *nasomaculatus* cytb as a wrongly classified element to its training class, but no misclassification was detected in the COI training classes.

BLOG recovered complete assignment for all the samples of *O*. *gazella* except for cytb sequence [GenBank:JX567271] because of an ambiguity in the identified diagnostic site for *O*. *gazella* (site 14259 in genome [GenBank:JN632678]). From a forensic perspective, this sample cannot be excluded from *O*. *gazella*.

#### Bovidae: Bovinae: Reduncini

This is a tribe of grazers associated with marshes and waterlands. It is composed of three genera: the monotypic *Pelea*, *Redunca* and *Kobus*. Of the six *Kobus* species, only the common waterbuck *K*. *ellipsiprymnus ellipsiprymnus* inhabits South Africa, and was found as a substitution for other game. The waterbuck displays parapatric geographic distribution with the defassa waterbuck *K*. *e*. *defassa* with an overlap in eastern Africa, where hybridization has been reported [52].We estimated the K2P distance between *K*. *ellipsiprymnus* and *K*. *kob* + *K*. *vardoni* as the latter is internal to the *K*. *kob* cluster for cytb sequences (Figure S1j in Additional file 3). Interspecific COI K2P distance was estimated using only *K*. *vardonii* because of the unavailability of *K*. *kob*.

The phylogenetic reconstructions in Figure S1j,k in Additional file 3 seem to reflect geographic origin. BLOG identified three classes within *K*. *ellipsiprymnus* with full correspondence with the cytb phylogenetic tree. Higher resolution is obtained with BLOG than with the ML tree for *K*. *vardoni* and *K*. *kob*.

#### Bovidae: Antilopinae: Cephalophini

This is a tribe of small antelopes with small horns know as duikers, which mostly inhabit forests. The common duiker was found in our samples as a substitute for springbok. This duiker species is ubiquitous in the sub-Saharan African continent, being absent only in rainforest areas and the horn of Africa. *Sylvicapra* was referred as monotypic [53] but recently shown to cluster within the giant *Cephalophus* duikers (*C*. *dorsalis*, *C*. *jentinki*, *C*. *silvicultor* and *C*. *spadix*) when using a multilocus phylogenetic approach [54], thus making *Cephalophus* paraphyletic. Similar results were obtained with whole mitochondrial sequence information by Hassanin *et al*. [40].

Johnston *et al*. [27] detected COI genetic distances below 3% between some *Cephalophus* species for their recent speciation processes estimated at ≤ one million years ago [54]. The interspecific distances for the common duiker shows an earlier speciation event, estimated at approximately 5.6 million years ago [27] (Figure 1).

The phylogeographic structure may account for the large cytb intraspecific distance observed in the common duiker (Figure 1a).

BLOG indicated 100% assignment to all training classes, identified the paraphyletic *C*. *callipygus* as a single class (Figure S1l in Additional file 3), and assigned the test sample to the common duiker class. In COI data analysis, one *C*. *ogilbyi* training element was recognized as a false positive due to limited information in the dataset, because of its recent evolutionary history [27]. The test sample remained unassigned because of an ambiguity in a diagnostic site. In forensic practice, this sequence cannot be excluded from ‘common duiker’ class (Figure S1m in Additional file 3).

#### Giraffidae

This family is composed of long-necked giraffes and okapis. The nine known *Giraffa camelopardalis* subspecies are widely distributed on the continent. The southern African *G*. *c*. *giraffe* is the only subspecies found in South Africa. Giraffe was found as a substitute for kudu biltong.

Brown *et al*. [55] and Hassanin *et al*. [56] studied the phylogeography of *Giraffa* using 1,143 and 1,765 bp mtDNA fragments respectively, encompassing cytb. Both studies identified strong phylogeographic structure in the continent. Brown *et al*. [55] argued that these subspecies should be considered separate species. The intraspecific maximum genetic distance in giraffes is unmatched (Figure 1a).

Our phylogenetic reconstruction shows the Angolan giraffe *G*. *c. angolensis* (Angola and Namibia) and the southern African giraffe (South Africa and Zimbabwe) sequences in a single cluster as a result of insufficient information in our shorter cytb fragment. There is insufficient information in this fragment to identify subspecies (Figure S1n in Additional file 3). BLOG found false negative and/or false positives in all training classes except for *G*. *c*. *peralta*, *G.c. reticulata* and *G.c. antiquorum* and the test sample is assigned only at the species level.

#### Suidae

Suidae is a family composed of pigs. The warthogs *Phacochoerus* are widely distributed sub-Saharan African wild pigs, represented by two disjunct subspecies: the common (*P*. *africanus africanus*) and the Ethiopian warthog (*P*. *a*. *aethiopicus*).

The two samples labeled warthog were correctly labeled whereas domestic pork was identified in an ostrich droë wors.

The monophyly of African pigs [57] (*Phacochoerus*, *Hylochoerus* and *Potamochoerus*) was not maintained when using the short cytb fragment used in this study. Therefore we used complete cytb sequence information recovered from GenBank along with our partial cytb fragments, applying partial deletion for missing data for phylogenetic reconstruction (Figure S1o in Additional file 3).

The two clusters observed for the common warthog correspond to geographic distribution. Continental phylogeographic structure was reported for this species with D-loop [58].

The phylogenetic reconstruction also shows an unusual clustering of the entries for *Sus celebensis* [GenBank: AY534298.1], *Potamochoerus porcus* [GenBank:AY534299.1] and *Potamochoerus larvatus* [GenBank:AY534300.1]. These are likely erroneous submissions. BLOG was run using the short cytb fragment dataset. Assignment classes for the test sequences were defined with 100% efficiency, no false positives or unassigned elements were detected in any species- defined class.

#### Equidae

This Perissodactyla family is composed of horses, donkeys and zebras. One sample labeled ‘zebra’ was indeed the mountain zebra *E. zebra*, whereas Burchell’s zebra and horse were found as substitutes for other game.

The study of the phylogenetic relationship of *Equus* species was been hampered by recent speciation processes, incomplete lineage sorting and introgression. Steiner *et al*. [59] resolved the phylogenetic relationship between zebra species using two mtDNA and 20 nuclear genes. Approximate tree topologies were recovered by our cytb and COI trees (Figure S1p,q in Additional file 3). Higher bootstrap supports were obtained for COI, but cytb can distinguish the two subspecies of cape mountain zebra *E* . *z*. *zebra* and Hartmann’s mountain zebra *E*. *z*. *hartmannae* that inhabit South Africa and Namibia, respectively. The tree in Figure S1p in Additional file 3 shows our sample #27 as Hartmann’s zebra. BLOG showed 100% efficiency to class (species) assignment.

The mountain zebra *E*. *zebra* suffered from severe reduction in population size in South Africa. The Mountain Zebra National Park hosted 19 mountain specimens at the time of its foundation in 1935. By then, only five other remnant populations were known in South Africa, where, unsurprisingly, strong population structure was detected using D-loop and microsatellites [60]. By contrast, the plains zebra showed no evidence of population structure [61].

#### Macropodidae

Kangaroo species were found as substitutes for six other game species. The low similarity of [GenBank:JX567266] to *M*. *robustus* is due to sequence ambiguities. However BLOG shows this sample as unassigned and all assignment classes (species) are defined with 100% efficiency. COI sequences were obtained both with universal and *Macropus*-specific primers designed in this study. Because of the currently limited COI entries for Macropodidae, identification with COI is only valid at the genus level, and four samples remained unassigned. The similarity of [GenBank:JX567041] to *Lagorchestes hirsutus*, a wallaby species (Table S2 in Additional file 2) is a clear example of anerroneous result because of incomplete information in databases.

#### Aves: Struthioniformes: Struthionidae

Ostrich was mostly found substituted (76% samples), by beef, kangaroo and other game (Table 2).

Population genetic data are only available for the mtDNA control region, which elucidated continental phylogeographic structure [62].

## Conclusions

### The practical problem of species delimitations

In forensics, it is important to provide an indication of the reliability of identification. It is beyond the scope of this paper to discuss the problems of species classification but rather we shall evaluate the methods of assignment for their application to wildlife or food forensics. All sequences were identified at subspecies, species or genera level for their high similarity to either vouchers or multiple entries in databases, monophyly with high bootstrap support, or full assignment to defined classes (subspecies, species, genus). The consistency in results obtained with methods based on different theoretical foundations demonstrates the reliability of the identifications.

The combined application of methods highlighted each of their strengths and weaknesses. Possibly the most important limitation affecting all methods is the incomplete within-species and taxa representation in databases. The African continent displays the highest diversity of ungulates on the planet, yet many species are poorly represented in databases. Phylogeographic structure at continental level and connectivity between east and southern Africa is common to many taxa because of the similar effects of Pleistocene climatic changes (see [63] and references therein). Most of these studies were conducted with the faster mutating control region. The geographic origin of samples on a large scale can still be inferred using cytb or COI fragments. In South Africa, the translocation of fauna is a natural consequence of the increasing ranching and private nature reserve activities. The species that are most frequently transferred outside their natural geographic limits are eland, gemsbok, blesbok, Burchell’s zebra and impala [64]. Therefore, geographic origin should be considered with caution for species with local population structure.

Incomplete geographic sampling of a species would determine underestimation of within-species genetic variation, and recent speciation processes would reflect shallow genetic distances between species, and often overlapping within-species variation. The latter case was evidenced for the South African and Tanzanian bushbucks. However, phylogenetic analysis allows for identification of these ‘overlapping’ forms, which show reciprocal monophyly.

Overlapping inter-intra species variation along with incomplete lineage sorting and absence of reciprocal monophyly was detected in *Connochaetes* (gnous), and in *Damaliscus pygargus*. D-loop seems to be more informative for both groups [45,47] (although paraphyly was described for blue wildebeest [47]). In addition, hybridization has been reported. Certainty can therefore be achieved at genus level for wildebeest in South Africa.

In general, similar results were recovered by the ML phylogenies and BLOG. The efficiency of BLOG is more reliant on the availability of large reference data. Nevertheless, its performance in limiting reference data conditions was similar to that of the phylogenetic approach. An exploratory phylogenetic analysis prior to the application of character-based methods would be advisable in the light of possible erroneous submissions to databases. Our phylogenetic analysis detected anomalous submissions to GenBank for Tragelaphini, Hippotragini and Suidae. BLOG showed full assignment for all our test sequences to predefined classes of species or even subspecies. Some elements identified as ‘unassigned’ for Alcelaphini, Cephalophini and Macropus required a ‘non-exclusion’ category applying forensic criteria. For the erroneous GenBank submission identified with ML, BLOG misidentified the erroneous sequence for Hippotragini cytb and did not detect COI classes with wrong elements. BLOG outperformed ML in the identification of Cephalophini cytb classes.

### Technical recommendations

In several mixtures it was not possible to generate readable sequences with both cytb and COI fragments (for example, lamb was detected with COI preferentially over beef and ostrich). Differential affinity for different species might explain these results. We obtained DNA from two to four different approximately 1 mm^2^ dissected fragments per sausage and therefore the presence of additional species cannot be discarded. A technical approach such as next generation sequencing would allow identifying other ‘alleles’ in the samples. A more affordable, though laborious, option is the standard PCR approach followed by Sanger sequencing followed by BLAST and phylogenetic analysis. A probe-based approach is not recommended at present for wildlife forensic applications because of the vast volume of uncovered genetic diversity on the continent.

### Commercial, political and religious implications of our results

The implications of our results have direct impact on the sensitivity of consumers. The avoidance of pork has serious religious connotations for the local Jewish and Muslim communities. The discovery of game substituted by kangaroo has an important social impact in Namibia as the local regulations do not support imported products being supplied to state institutions [65]. The consumer should have the right to choose over correctly provided information. A change in the labeling system to indicate generic ‘game’ should be clearly distinguished from appropriately indicated domestic or foreign species.

## Endnotes

^a^ Burchell’s zebra (*Equus quagga burchelli*) is a variety of the plains zebra *E*. *quagga*. It is often referred to as *E*. *burchelli*, or even *E*. *burchellii quagga*.

## Abbreviations

BLAST: Basic Local Alignment Search Tool; BOLD: Barcode of Life Data Systems; Bp: Base pairs; CITES: Convention on International Trade in Endangered Species of Wild Fauna and Flora; COI: cytochrome c oxidase subunit I; cytb: cytochrome b; IUCN: International Union for Conservation of Nature; K2P: Kimura-2-parameter; ML: maximum likelihood; mtDNA: mitochondrial DNA; PCR: polymerase chain reaction

## Competing interests

The authors declare that they have no competing interests.

## Authors’ contributions

MED proposed the experimental design and performed the data analysis; MED and SD wrote the manuscript; KWC, SD and MED performed the sampling; KWC, EA and DC generated the data; DC proposed the original idea. All authors read and approved the manuscript.

## Supplementary Material

Additional file 1: Table S1 GenBank accession numbers of cytb and COI sequences used in the estimation of K2P shown in Figure 1, phylogenetic analyses and BLOG analysis shown in Table S2 in Additional file 2. In bold: reference sequences generated in this study. Click here for file

Additional file 2: Table S2 Summary of species identification results for cytb and COI sequences obtained from commercial meat products. The maximum sequence similarity with GenBank entries is provided in % identity, along with the phylogenetic tree bootstrap support for the species or subspecies clusters. BLOG assignments to defined classes: 100; U: unassigned elements. Click here for file

Additional file 3 Consensus Maximum Likelihood trees constructed using Kimura 2-parameter model and 1,000 bootstrap replicates. Branches corresponding to partitions reproduced in less than 50% bootstrap replicates are collapsed; bootstrap support is shown on the nodes. Substitutions are shown in red with the sample ID and species indicated by the commercial label. Species names are kept as the original submissions to GenBank. When known, the specimen geographic origin is indicated: Aby: Abyssinia; BNP: Bontebok National Park; Cam: Cameroon; CAR: Central African Republic; Chad: Chad; E Africa: East Africa; Gui: Guinea; Ken: Kenya; Ma: Mali; MZNP: Mountain Zebra National Park; Niga: Nigeria; Niger: Niger; NZG: National Zoological Gardens; SA: South Africa; Sau: Saudi Arabia; So: Somalia; Tan: Tanzania; Uga: Uganda; Zam: Zambia; Zim: Zimbabwe. Colored blocks indicate clusters defined with 100% assignment efficiency by BLOG. Substitutions are shown in red, food samples ID can be found in Table S2 in Additional file 2. **Figure S1a** Aepycerotyni and Antilopini COI sequences. ML Tree Log = -2234.00. **Figure S1b.** Alcelaphinae cytb sequences. ML Tree Log = -1510.45 . ** element not assigned to any class. **Figure S1c.** Alcelaphini COI sequences. Tree Log L = -1252.20. **Figure S1d.** Caprini COI sequences. Tree Log L = -2008.41. **Figure S1e.** Bovini cytb sequences. Tree Log L = -1134.75. **Figure S1f.** Tragelaphini cytb sequences. * erroneous submission to GenBank. *T*. *eurycerus*: JN632703, AF036276, AF022065; *T*. *spekii*: EF536357, AJ222680; *T*. *buxtoni*: AY667215-216; Tree LogL = -2153.35. BLOG recovered classes with fully assigned (either test or training) elements, which are indicated with colored boxes. Training classes defined for all other species contained 100% assigned elements. **Figure S1g.** Tragelaphini COI sequences. Tree LogL = -2146.42. BLOG recovered classes with fully assigned elements, which are indicated with colored boxes. Uncolored boxes: other defined training classes with correct classified elements. **Figure S1h.** Hippotragini cytb sequences. Tree LogL = -1020.96. * erroneous submission; ** BLOG wrong classified element. *Oryx beisa* GenBank entries are JN632676.1, DQ138192.1-210.1; HM209249.1. **Figure S1i.** Hippotragini COI sequences. Tree LogL = -1874.32. * possible erroneous submission. **Figure S1j.***Reduncinae* cytochrome b sequences. Tree LogL = -1387.71. BLOG recovered classes with fully assigned (either test or training) elements, which are indicated with colored boxes. Training classes defined for all other species contained 100% assigned elements. **Figure S1k.***Reduncinae* COI sequences. Tree LogL = -1501.5. BLOG recovered classes with fully assigned (either test or training) elements, which are indicated with colored boxes. Training classes defined for all other species contained 100% assigned elements. **Figure S1l.** Cephalophinae cytb sequences. Tree LogL = -2206.5. Cephalophinae cytb GenBank accession numbers for *Philatomba maxwelli* are JN632885, AF096629.1, AF153894.1; for *P*. *monticola* are JN632686, JN632687, AF153891.1- 893.1; for *Cephalophus dorsalis* are JN632615.1; AF153884.1, AF091634; for *C*. *silvicultor* AF153898.1, JN632622.1; for *C*. *spadix* are AF153899.1, JN632623.1; for *C*. *callipygus* are AF153885-86, JN632613-14; for *C*. *leucogaster* are JN632617.1, AF153889.1; for *C*. *nigrifrons* are JN632619.1, AF153886.1; for *C*. *rufilatus* are JN632621.1, AF153901.1, for *C*. *natalensis* are JN632618.1, AF153890.1, for C. *harveyi* are FJ959388.1, AF153887.1 and for *C*. *adersi* are JN632611.1, AF153883.1. All classes defined by BLOG correspond to nominated species. In colored boxes: the class for the test sample #53, *Sylvicapra grimmia*, and the separate classes identified for *C*. *callipygus* and *C*. *ogylbyi*. **Figure S1m.** Cephalophinae COI sequences. Cephalophinae cytb GenBank accession numbers for *Cephalophus dorsalis* are JN632615.1; HQ644091.1, HQ644092.1; GQ144507.1- 514.1; for *C*. *nigrifrons* are GQ144446.1-450.1; *C*. *rufilatus* are JN632621.1, HQ644111.1; for *C*. *leucogaster* are JN632617.1, GQ144515.1-521.1; HQ644095.1-968.1; for *C*. *niger* are HQ644105-106.1; for *C*. *callipygus* cluster **(1)** are JN632612.1, GQ144485.1, GQ144486.1, GQ144491.1, GQ14492.1, GQ144498.1, GQ144499.1, GQ144504.1, HQ644089.1, HM144023, HM144025; for *C*. *callipygus* cluster **(2)** are JN632614.1, GQ144483.1, GQ144487-489.1, GQ144493-495.1, GQ144500-503.1, GQ144505.1, HQ644090.1, HQ644109.1; for *C*. *adersi* are HQ644086.1, HQ644087.1; for *Philatomba monticola* cluster (1) are JN632887, HQ644101.1; for *P*. *monticola* cluster (2) JN632686, GQ144522-533.1, GQ144535.1, GQ144540.1, GQ144543.1, GQ144544.1; for *Philatomba maxwelli* are JN632685, HQ644099.1, HQ655100.1. Tree LogL = -3675.85. ** BLOG not classified sequences; *** BLOG erroneously classified sequence. Colored box indicates the BLOG class defined class for *S*. *grimmia*. **Figure S1n**. Giraffidae cytb sequences. The GenBank accession number for cluster (1) are EU088326.1- EU088328.1, EF442273.1; for cluster (2) are EF442274.1, EU088317.1-EU088317.1; for cluster (3) are JN632645.1, EF442265.1-EF442268.1; for cluster (4) are EU088319.1, EU088320.1, EU088322.1- EU088325.1, EU088334.1; for cluster (5) are AY534342.1, EU08331.1, EU088333.1; for cluster (6) DQ470781.1, EF442269.1, EF442270.1, EF442271.1 from Limpopo, South Africa, EU088329.1, EU088330.1 EU088332.1, EU088335.1-EU088344.1; for cluster (7) are EU088345.1-EU088351.1, EF442263.1, EF442264.1, AP003424.1, NC_012100.1; for cluster (8) are EU088352.1, AF181470.1, AY121933.1. Tree LogL = -879.10. **Figure S1o.** Suidae whole cytb sequences (1140 bp). The bootstrap values were inferred from 1,000 replicates. Tree LogL = -6959.31. The GenBank accession numbers for cluster (1) are GQ338962.1, AY534297.1, AJ314554.1, AJ314555.1, AM492658-662; for cluster (2) are GQ338963.1, AJ314553.1; for cluster (3) are NC_000845, NC_012095.1, NC_014692.1, AY692029.1, for cluster (4) are AM492663-665, GQ338964.1. * erroneous submission to GenBank. Assignments (classes) defined by BLOG with 100% efficiency are shown with colored boxes. **Figure S1p**. Equidae cytb sequences. Tree Log L = -1,000.63. The GenBank accession numbers for *Equus assinus* are FF18884, NC001788, FJ428492, FJ428526 and *E*. *a*. *somalicus* FJ428508. BLOG assignment classes are shown in colored blocks. **Figure S1q**. Equidae COI sequences. Tree LogL = -1870.39. **Figure S1r**. Macropodidae cytb sequences. Tree LogL = -1665.38. BLOG classes are shown in colored boxes. * unassigned sample. Click here for file
