# Supplementary material for: Where is the game? Wild meat products authentication in South Africa: a case study
Source: Investig Genet. 2013 Mar 1;4:6. doi: 10.1186/2041-2223-4-6 (PMC3621286; doi:10.1186/2041-2223-4-6)
Supplement: Additional file 1: Table S1 — GenBank accession numbers of cytb and COI sequences used in the estimation of K2P shown in Figure 1, phylogenetic analyses and BLOG analysis shown in Table S2 in Additional file 2. In bold: reference sequences generated in this study. [file 2041-2223-4-6-S1.doc]

**Supplementary Table 1. {Au query: Please check the highlighted sections for accuracy.}**

| **Group** | **cytb** | **COI** |
| --- | --- | --- |
| Bovinae: Aepycerotinae | AF022056.1, AF034966.1, AF036289.1, AF301730.1, AF301729.1, AF301728.1, AF301727.1, AF301726.1, AF301724.1, AF301722.1, AF301721.1, AF301720.1, AF301719.1, AF301718.1, AF301717.1, JN632668, JN632669, JN632668, JN632669, AF301716.1, AF301715.1, AF301713.1, JN632592.1 | HQ603134.1, HQ603135.1, HQ603136.1, HQ603137.1, HQ603138.1, HQ603140.1, HQ603141.1, HQ603142.1, HQ603143.1, JN632592.1, JN632668, JN632669 |
| Bovinae: Antilopini | AF022054.1, JN632596.1, AF036281.1, JN632637.1, JN632653.1, JN632680.1, JN632693.1 | **JX436990-92**, JN632596.1, JN632637.1, JN632653.1, JN632680.1, JN632693.1 |
| Bovinae: Alcelpahinae *Alcelaphus sp* | AF034967.1, JN632593.1, JN632594.1, AF016636.1, AF016640.1, AF300932.1, AJ222681.1, FJ785383.1, FJ785382.1, AF300933.1-42.1, DQ470785.1, DQ470784.1, AF028822.1 | HQ603091.1, HQ603088.1-90.1, HQ603087.1, JN632593.1, JN632594.1 |
| Bovinae: Alcelpahinae *Connochaetes sp* | AF016637.1, AF016638.1, AF034969.1, DQ470801.1, DQ470802.1, FJ785393.1, FJ785394.1, HM209246.1, HM209247.1, JN632626.1, JN632627.1, JN632628.1, AF016637.1 | **JX436976-80**, HQ603109.1-22, JN632626.1, JN632627.1, JN632628.1, JQ690393.1 |
| Bovinae: Alcelpahinae *Damaliscus pygargus* | **JX436997-7000**, FJ207530.1, AF036287, AF016639.1 | **JX436981-89,** FJ207530.1 |
| Bovinae: Caprini | NC_001941, HM236176.1, HM236175.1, HM236188.1, FJ207522.1, FJ207538.1, FJ207526.1, GU068049.1, NC_007441.1, FJ207533.1 | NC_001941, HM236176.1, HM236175.1, HM236188.1, FJ207522.1, FJ207538.1, FJ207526.1, GU068049.1, NC_007441.1, FJ207533.1 |
| Bovidae: Bovini | NC_005863, V00654, AF492351, AF492350.1, EU177868.1, NC_005971, JN632606.1, JN632602.1, EF536353.1, JQ235508.1, EF536351.1, JN632607.1 | NC_005863, V00654, AF492351, AF492350.1, EU177868.1, NC_005971, JN632606.1, JN632602.1, EF536353.1, JQ235508.1, EF536351.1, JN632607.1 |
| Bovinae: Tragelaphini | AF022057.1, AF022063.1, AF022065.1, AF022066.1, AF022067.1, AF030266.1, AF030269.1, AF036276.1, AF036277.1, AF036278.1, AF036280.1, AF091633.1, AJ222680.1, DQ470778.1, EF536354.1, EF536356.1, EF536357.1, FJ785378.1, FJ785379.1, HQ641312.1, HQ641313.1, JN632702.1, JN632703.1, JN632704.1, JN632705.1, JN632706.1, JN632707.1, JN632708.1, L13793.1, L13794.1 | **JX436993, JX436994**, EF536354.1, EF536356.1, EF536357.1, HQ603157.1, HQ603161.1, HQ603162.1, HQ603169.1, HQ603170.1, HQ603171.1, HQ603172.1, HQ603173.1, HQ603174.1, HQ644120.1, JN632702.1, JN632704.1, JN632705.1, JN632706.1, JN632707.1, JN632708.1, JQ690384.1 |
| Bovinae: Hippotragini | AF036286.1, AF249973.1, AF249973.1, AJ222685.1, DQ138192.1 -210.1, FJ937660.1, HM209249.1, JN632676.1, JN632677.1, JN632678.1, JN632679.1, JN869312.1, NC_016422.1, | **JX436995**, JF444371.1, JF444372.1, JF444372.1, JN632591.1, JN632647.1, JN632648.1, JN632676.1-79.1, JN869311.1, JN869312.1, NC_016422.1 |
| Bovinae: Reduncini | AF022055, AF022059, AF036284, AF052939, AF096619, AF096620-28, AJ222686, AY534347, DQ470800, HQ641319, JN632651, JN632652, JN632652, JN632684, JN632693, JN632694, JN632695 | JN632651.1, HQ603160.1, HQ603158.1, HQ603159.1, JQ690391.1, JQ690383.1, JQ690383.1, JQ690387.1, JN632652.1, JN632694.1, JN632684.1, JN632695.1 |
| Bovinae: Cepalophini | AF091634, AF096629, AF153883, AF153884-905, AJ222684, DQ470779, DQ470780, FJ785376, FJ785377, FJ959388, JN632611, JN632612, JN632613, JN632614, JN632615, JN632616, JN632617, JN632618, JN632619, JN632620, JN632621, JN632622, JN632623, JN632685, JN632686, JN632687, JN632701 | GQ144483-533, GQ144535, GQ144540, GQ144542-544, GQ144546, GQ144547-550, HM144023, HM144025, HQ644086-119, JN632612, JN632613, JN632614, JN632615, JN632616, JN632618, JN632619, JN632620, JN632621, JN632622, JN632685, JN632686, JN632687, JN632701 |
| Giraffidae | AF181470.1, AP003424, AY121993.1, EF442263-274, EU088317-352.1, JN632645, JN632674.1, NC012100, X56287 | NC012100, JN632645.1, JF444302.1, FJ958342.1, JN632674.1, JN632674.1 |
| Suidae | AJ314547-551, AM492660, AM492663, AM492664, AM492665, AY534297-303, AY534348, AY920905, AY920909, DQ315602, DQ409327, DQ470796-799, EF545571, EF545592, FJ237000, FJ785389, FJ785390, GQ338964, GQ338966, GQ338968, GQ338969, HQ634692, HQ634693, HQ680407, JN632682, JN632688, NC000845, NC008830, Z50090 | DQ409327.1, EU333163.1, FJ237000.1, GQ144632.1, GQ144632.1, GQ144633.1, GQ144634.1, GQ144635.1, GQ144636.1, GQ144637.1, GQ144638.1, JN632682.1, JN632688.1, JQ690385.1, NC_000845 |
| Equidae | **JX567002-7005**, AY534349, DQ470804, DQ470805, FF718884, FJ428492, FJ428508, FJ428526, FJ785391, FJ785392, JF718883, JF718885, JF718886, JF718887, JF718888, JF718889, JF718890, JN398385, JN398399, JN398402, JN398403, JN398405, JN398440, JN398455, JX312717, JX312718, JX312719, JX312720, JX312721, JX312728, JX312729, JX312733, NC001640, NC001788, X56282 | **JX436996, JX566994-7001,** AP012267, AP012268, AP012269, AP012271, EF568640, EF568729, HM118851, JN398385, JN398399, JN398402, JN398403, JN398405, JN398440, JN398455, JQ690388, JX312717, JX312718, JX312719, JX312720, JX312720, JX312721, JX312728, JX312729, JX312733, JX436996, JX566994 -7001, NC001640, NC001788, X97337 |
| Macropodidae | AY099267, AY099270, AY099271, AY099272, AY099280, AY099281, AY237226, AY237227, AY237228, AY237229, EF368023, EF368024, EF368025, EF368026, EF368027, EF368028, EF368029, EF368030, JN003400, NC_001794, U87136, U87137, Y10524 | NC_001794.1, Y10524.1, JQ042183.1, AB241056.1, JQ042165-187.1, AJ304826.1, AJ639872.1, Z29573.1 |
| AVES  *Struthio camelus* | AF338715.1, NC_002785, NC_001323.1, NC_002783, NC_013978.1 | AF338715.1, NC_002785, NC_001323.1, NC_002783, NC_013978.1 |

COI: cytochrome c oxidase subunit I; cytb: cytochrome b;
