# Supplementary material for: Where is the game? Wild meat products authentication in South Africa: a case study
Source: Investig Genet. 2013 Mar 1;4:6. doi: 10.1186/2041-2223-4-6 (PMC3621286; doi:10.1186/2041-2223-4-6)
Supplement: Additional file 2: Table S2 — Summary of species identification results for cytb and COI sequences obtained from commercial meat products. The maximum sequence similarity with GenBank entries is provided in % identity, along with the phylogenetic tree bootstrap support for the species or subspecies clusters. BLOG assignments to defined classes: 100; U: unassigned elements. [file 2041-2223-4-6-S2.doc]

Supplementary Table 2.

|  |  |  |  |  | |  |  |  |  |  |  |  |  | |  | |  | | |
| --- | --- | --- | --- | --- | --- | --- | --- | --- | --- | --- | --- | --- | --- | --- | --- | --- | --- | --- | --- |
|  |  |  |  | **Results** | |  |  |  |  |  |  |  |  | |  | |  | | |
| **Samples** | |  |  | **cytb** | |  |  |  |  | **COI** |  |  |  | |  | |  | | |
| **Lab. label** | **Commercial label** | **Meat process** | **Expected species** | **GenBank Accession** | **Similar to** | | **Max. identity** | **ML boots (%)** | **BLOG** | **GenBank Accession** | **Similar to** | **Max. identity** | **ML boots (%)** | | **BLOG** | | |  | |
| 1 | Kudu | biltong | *Tragelaphus strepsiceros* | JX567157 | | *Ovis aries* | 99% | 99 (*) | 100 | JX567005 | *Ovis aries* | 100% | 79 | | 100 | |  | | |
| 2 | Kudu | biltong | *Tragelaphus strepsiceros* | JX567158 | | *Alcelaphus buselaphus* | 100% | 78 | 100 | JX567006 | *Alcelaphus buselaphus* | 99% | 92 (S) | | 100 | |  | | |
| 3 | Beef | biltong | *Bos taurus* | JX567159 | | *Bos taurus* | 100% | 94 | 100 | JX567007 | *Bos taurus* | 100% | 99 | | 100 | |  | | |
| 4 | Game | biltong | *any game species* | JX567160 | | *Oryx gazella* | 100% | 100 | 100 | JX567008 | *Oryx gazella* | 100% | 99 | | 100 | |  | | |
| 5 | Beef | biltong | *Bos taurus* | JX567161 | | *Bos taurus* | 100% | 94 | 100 | JX567009 | *Bos taurus* | 100% | 99 | | 100 | |  | | |
| 6 | Kudu | smoked | *Tragelaphus strepsiceros* | JX567162 | | *Tragelaphus strepsiceros* | 97% | 92 | 100 | JX567010 | *Tragelaphus strepsiceros* | 98% | 98 | | 100 | |  | | |
| 7 | Ostrich | smoked | *Struthio camelus* | JX567163 | | *Tragelaphus strepsiceros* | 97% | 92 | 100 | JX567011 | *Tragelaphus strepsiceros* | 98% | 98 | | 100 | |  | | |
| 8 | Kudu | droë wors | *Tragelaphus strepsiceros* | JX567164 | | *Tragelaphus strepsiceros* | 96% | 92 | 100 | JX567012 | *Tragelaphus strepsiceros* | 97% | 98 | | 100 | |  | | |
| 9 | Kudu | biltong | *Tragelaphus strepsiceros* | JX567165 | | *Oryx gazella* | 100% | 100 | 100 | JX567013 | *Oryx gazella* | 100% | 99 | | 100 | |  | | |
| 10 | Springbok | biltong | *Antidorcas marsupialis* | JX567166 | | *Antidorcas marsupialis* | 99% | 99 | 100 | JX567014 | *Antidorcas marsupialis* | 99% | 99 | | 100 | |  | | |
| 11 | Springbok | droë wors | *Antidorcas marsupialis* | JX567167 | | *Antidorcas marsupialis* | 100% | 99 | 100 | JX567015 | *Antidorcas marsupialis* | 99% | 99 | | 100 | |  | | |
| 12 | Eland | biltong | *Tragelaphus oryx* | JX567168 | | *Oryx gazella* | 100% | 100 | 100 | JX567016 | *Oryx gazella* | 100% | 99 | | 100 | |  | | |
| 13 | Gemsbok | biltong | *Oryx gazella* | JX567169 | | *Tragelaphus strepsiceros* | 100% | 92 | 100 | JX567017 | *Tragelaphus strepsiceros* | 99% | 98 | | 100 | |  | | |
| 14 | Ostrich | droë wors | *Struthio camelus* | JX567170 | | *Struthio camelus* | 99% | 100 | 100 | JX567018 | *Ovis aries* | 100% | 79 | | 100 | |  | | |
| 15 | Beef | biltong | *Bos taurus* | JX567171 | | *Bos taurus* | 100% | 94 | 100 | JX567019 | *Bos taurus* | 100% | 99 | | 100 | |  | | |
| 16 | Ostrich | biltong | *Struthio camelus* | JX567172 | | *Bos taurus* | 100% | 94 | 100 | JX567020 | *Bos taurus* | 100% | 99 | | 100 | |  | | |
| 17 | Kudu | biltong | *Tragelaphus strepsiceros* | JX567173 | | *Oryx gazella* | 100% | 100 | 100 | JX567021 | *Oryx gazella* | 100% | 99 | | 100 | |  | | |
| 18 | Eland | biltong | *Tragelaphus oryx* | JX567174 | | *Tragelaphus oryx* | 99% | 91 | 100 | JX567022 | *Tragelaphus oryx* | 99% | 98 (S) | | 100 (S) | |  | | |
| 19 | Gemsbok | biltong | *Oryx gazella* | JX567175 | | *Damaliscus pygargus* | 99% | 88 | 100 | JX567023 | *Damaliscus pygargus* | 100% | 99 | | 100 | |  | | |
| 20 | Springbok | biltong | *Antidorcas marsupialis* | JX567176 | | *Oryx gazella* | 100% | 100 | 100 | JX567024 | *Oryx gazella* | 100% | 99 | | 100 | |  | | |
| 21 | Springbok | biltong | *Antidorcas marsupialis* | JX567177 | | *Antidorcas marsupialis* | 100% | 99 | 100 | JX567025 | *Antidorcas marsupialis* | 99% | 99 | | 100 | |  | | |
| 22 | Kudu | droë wors | *Tragelaphus strepsiceros* | JX567178 | | *Tragelaphus strepsiceros* | 99% | 92 | 100 |  |  |  |  | |  | |  | | |
| 22 |  |  |  | JX567179 | | *Oryx gazella* | 99% | 100 | 100 |  |  |  |  | |  | |  | | |
| 23 | Ostrich | droë wors | *Struthio camelus* | JX567180 | | *Bos taurus* | 100% | 94 | 100 | JX567026 | *Bos taurus* | 100% | 99 | | 100 | |  | | |
| 24 | Springbok | biltong | *Antidorcas marsupialis* | JX567181 | | *Bos taurus* | 98% | 94 | 100 | JX567027 | *Oryx gazella* | 98% | 99 | | 100 | |  | | |
| 24 |  |  |  | JX567182 | | *Oryx gazella* | 100% | 100 | 100 |  |  |  |  | |  | |  | | |
| 25 | Springbok | biltong | *Antidorcas marsupialis* | JX567183 | | *Tragelaphus strepsiceros* | 99% | 92 | 100 | JX567028 | *Tragelaphus strepsiceros* | 99% | 98 | | 100 | |  | | |
| 26 | Kudu | smoked | *Tragelaphus strepsiceros* | JX567184 | | *Oryx gazella* | 100% | 100 | 100 | JX567029 | *Oryx gazella* | 99% | 99 | | 100 | |  | | |
| 27 | zebra | smoked | *Equus zebra/E. q. burchellii* | JX567185 | | *Equus zebra* | 100% | **98 (N)** | 100 | JX567030 | *Equus zebra* | 99% | 97 | | 100 | |  | | |
| 28 | Beef | biltong | *Bos taurus* | JX567186 | | *Bos taurus* | 100% | 94 | 100 | JX567031 | *Bos taurus* | 100% | 99 | | 100 | |  | | |
| 29 | Kudu | biltong | *Tragelaphus strepsiceros* | JX567187 | | *Bos taurus* | 100% | 94 | 100 | JX567032 | *Bos taurus* | 100% | 99 | | 100 | |  | | |
| 30 | Gemsbok | biltong | *Oryx gazella* | JX567188 | | *Bos taurus* | 100% | 94 | 100 | JX567033 | *Bos taurus* | 100% | 99 | | 100 | |  | | |
| 31 | Springbok | biltong | *Antidorcas marsupialis* | JX567189 | | *Bos taurus* | 100% | 94 | 100 | JX567034 | *Bos taurus* | 100% | 99 | | 100 | |  | | |
| 32 | Eland | biltong | *Tragelaphus oryx* | JX567190 | | *Bos taurus* | 99% | 94 | 100 | JX567035 | *Bos taurus* | 100% | 99 | | 100 | |  | | |
| 33 | Springbok | biltong | *Antidorcas marsupialis* | JX567191 | | *Tragelaphus strepsiceros* | 97% | 92 | 100 | JX567036 | *Tragelaphus strepsiceros* | 98% | 98 | | 100 | |  | | |
| 34 | Springbok | droë wors | *Antidorcas marsupialis* | JX567192 | | *Bos taurus* | 100% | 94 | 100 | JX567037 | *Bos taurus* | 100% | 99 | | 100 | |  | | |
| 34 |  |  |  | JX567193 | | *Connochaetes nou* | 100% | 72 | 100 | JX567038 | *Kobus ellipsiprymnus* | 98% | 94 | | 100 | |  | | |
| 35 | Kudu | biltong | *Tragelaphus strepsiceros* | JX567194 | | *Bos taurus* | 100% | 94 | 100 | JX567039 | *Bos taurus* | 100% | 99 | | 100 | |  | | |
| 36 | Gemsbok | biltong | *Oryx gazella* | JX567195 | | *Tragelaphus strepsiceros* | 97% | 92 | 100 | JX567040 | *Tragelaphus strepsiceros* | 98% | 98 | | 100 | |  | | |
| 37 | Kudu | droë wors | *Tragelaphus strepsiceros* | JX567196 | | *Macropus rufus* | 100% | 97 | 100 | JX567041 | *Lagorchestes hirsutus* | 88% | 100 (Mr) | | U | |  | | |
| 38 | Springbok | droë wors | *Antidorcas marsupialis* | JX567197 | | *Bos taurus* | 100% | 94 | 100 | JX567042 | *Bos taurus* | 100% | 99 | | 100 | |  | | |
| 39 | Springbok | biltong | *Antidorcas marsupialis* | JX567198 | | *Bos taurus* | 100% | 94 | 100 | JX567043 | *Bos taurus* | 100% | 99 | | 100 | |  | | |
| 40 | Springbok | biltong | *Antidorcas marsupialis* | JX567199 | | *Alcelaphus buselaphus* | 99% | 78 | U | JX567044 | *Alcelaphus buselaphus* | 99% | 92 (S) | | 100 | |  | | |
| 41 | Kudu | biltong | *Tragelaphus strepsiceros* | JX567200 | | *Alcelaphus buselaphus* | 99% | 78 | 100 | JX567045 | *Alcelaphus buselaphus* | 99% | 92 (S) | | 100 | |  | | |
| 42 | Eland | biltong | *Tragelaphus oryx* | JX567201 | | *Oryx gazella* | 100% | 100 | 100 | JX567046 | *Oryx gazella* | 100% | 99 | | 100 | |  | | |
| 43 | Kudu | droë wors | *Tragelaphus strepsiceros* | JX567202 | | *Bos taurus* | 99% | 94 | 100 | JX567047 | *Bos taurus* | 100% | 99 | | 100 | |  | | |
| 44 | Springbok | carpaccio | *Antidorcas marsupialis* | JX567203 | | *Connochaetes taurinus* | 100% | 66 (SA) | 100 | JX567048 | *Connochaetes taurinus* | 100% | 73 (SA) | | 100 (G) | |  | | |
| 45 | Kudu | carpaccio | *Tragelaphus strepsiceros* | JX567204 | | *Equus burchellii* | 99% | <50 (**) | 100 | JX567049 | *Equus burchellii* | 99% | 98 | | 100 | |  | | |
| 46 | Impala | carpaccio | *Aepyceros melampus* | JX567205 | | *Aepyceros melampus* | 99% | 99 | 100 | JX567050 | *Aepyceros melampus* | 99% | 100 | | 100 | |  | | |
| 47 | Warthog | carpaccio | *Phacochoerus africanus* | JX567206 | | *Phacochoerus africanus* | 99% | 99 (*) | 100 | JX567051 | *Phacochoerus africanus* | 99% | 99 | | U | |  | | |
| 48 | Gemsbok | mince | *Oryx gazella* | JX567207 | | *Tragelaphus strepsiceros* | 97% | 92 | 100 | JX567052 | *Tragelaphus strepsiceros* | 100% | 98 | | 100 | |  | | |
| 49 | Warthog | mince | *Phacochoerus africanus* | JX567208 | | *Phacochoerus africanus* | 97% | 99 (*) | 100 | JX567053 | *Phacochoerus africanus* | 99% | 99 | | U | |  | | |
| 50 | Springbok | mince | *Antidorcas marsupialis* | JX567209 | | *Antidorcas marsupialis* | 100% | 99 | 100 | JX567054 | *Antidorcas marsupialis* | 99% | 99 | | 100 | |  | | |
| 51 | Ostrich | mince | *Struthio camelus* | JX567210 | | *Antidorcas marsupialis* | 100% | 99 | 100 | JX567055 | *Antidorcas marsupialis* | 99% | 99 | | 100 | |  | | |
| 52 | Springbok | smoked | *Antidorcas marsupialis* | JX567211 | | *Aepyceros melampus* | 100% | 99 | 100 | JX567056 | *Aepyceros melampus* | 99% | 100 | | 100 | |  | | |
| 53 | Springbok | biltong | *Antidorcas marsupialis* | JX567212 | | *Sylvicapra grimmia* | 98% | 99 | U | JX567057 | *Sylvicapra grimmia* | 99% | 99 | | U | |  | | |
| 54 | Kudu | biltong | *Tragelaphus strepsiceros* | JX567213 | | *Tragelaphus strepsiceros* | 97% | 92 | 100 | JX567058 | *Tragelaphus strepsiceros* | 98% | 98 | | 100 | |  | | |
| 55 | Gemsbok | biltong | *Oryx gazella* | JX567214 | | *Oryx gazella* | 99% | 100 | 100 | JX567059 | *Oryx gazella* | 99% | 99 | | 100 | |  | | |
| 56 | Kudu | biltong | *Tragelaphus strepsiceros* | JX567215 | | *Bos taurus* | 100% | 94 | 100 | JX567060 | *Bos taurus* | 100% | 99 | | 100 | |  | | |
| 57 | Kudu | droë wors | *Tragelaphus strepsiceros* | JX567216 | | *Bos taurus* | 100% | 94 | 100 | JX567061 | *Bos taurus* | 100% | 99 | | 100 | |  | | |
| 58 | Ostrich | biltong | *Struthio camelus* | JX567217 | | *Struthio camelus* | 99% | 100 | 100 | JX567062 | *Struthio camelus* | 99% | 100 | | 100 | |  | | |
| 59 | Ostrich | carpaccio | *Struthio camelus* | JX567218 | | *Struthio camelus* | 99% | 100 | 100 | JX567063 | *Struthio camelus* | 99% | 100 | | 100 | |  | | |
| 60 | Springbok | carpaccio | *Antidorcas marsupialis* | JX567219 | | *Antidorcas marsupialis* | 100% | 99 | 100 | JX567064 | *Antidorcas marsupialis* | 99% | 99 | | 100 | |  | | |
| 61 | Ostrich | mince | *Struthio camelus* | JX567220 | | *Bos taurus* | 99% | 94 | 100 | JX567065 | *Bos taurus* | 100% | 99 | | 100 | |  | | |
| 62 | Kudu | biltong | *Tragelaphus strepsiceros* | JX567221 | | *Bos taurus* | 100% | 94 | 100 | JX567066 | *Bos taurus* | 100% | 99 | | 100 | |  | | |
| 63 | Kudu | droë wors | *Tragelaphus strepsiceros* | JX567222 | | *Bos taurus* | 100% | 94 | 100 | JX567067 | *Bos taurus* | 100% | 99 | | 100 | |  | | |
| 64 | Kudu | biltong | *Tragelaphus strepsiceros* | JX567223 | | *Kobus ellipsiprymnus* | 100% | 90 (SE) | 100 | JX567068 | *Kobus ellipsiprymnus* | 98% | 94 | | 100 | |  | | |
| 65 | Gemsbok | biltong | *Oryx gazella* | JX567224 | | *Oryx gazella* | 100% | 100 | 100 | JX567069 | *Oryx gazella* | 99% | 99 | | 100 | |  | | |
| 66 | Blue wildebeest | biltong | *Connochaetes taurinus* | JX567225 | | *Tragelaphus angasii* | 100% | 99 | 100 | JX567070 | *Tragelaphus angasii* | 99% | 100 | | 100 | |  | | |
| 67 | Kudu | droë wors | *Tragelaphus strepsiceros* | JX567226 | | *Macropus rufus* | 100% | 97 | 100 | JX567071 | *Macropus robustus* | 99% | 100 (Mr) | | U | |  | | |
| 68 | Springbok | droë wors | *Antidorcas marsupialis* | JX567227 | | *Bos taurus* | 100% | 94 | 100 | JX567072 | *Ovis aries* | 99% | 79 | | 100 | |  | | |
| 69 | Kudu | biltong | *Tragelaphus strepsiceros* | JX567228 | | *Oryx gazella* | 100% | 100 | 100 | JX567073 | *Oryx gazella* | 100% | 99 | | 100 | |  | | |
| 70 | Springbok | biltong | *Antidorcas marsupialis* | JX567229 | | *Connochaetes gnou* | 100% | 72 | 100 | JX567074 | *Connochaetes gnou* | 99% | 55 | | **100 (G)** | |  | | |
| 71 | Kudu | droë wors | *Tragelaphus strepsiceros* | JX567230 | | *Oryx gazella* | 100% | 100 | 100 | JX567075 | *Oryx gazella* | 99% | 99 | | 100 | |  | | |
| 71 |  |  |  |  | |  |  |  |  | JX567076 | *Alcelaphus buselaphus* | 97% | 92 (S) | | 100 | |  | | |
| 72 | Game | droë wors | *any game species* | JX567231 | | *Oryx gazella* | 100% | 100 | 100 | JX567077 | *Oryx gazella* | 99% | 99 | | 100 | |  | | |
| 72 |  |  |  | JX567232 | | *Tragelaphus strepsiceros* | 97% | 92 | 100 |  |  |  |  | |  | |  | | |
| 73 | Ostrich | fresh wors | *Struthio camelus* | JX567233 | | *Sus scrofa* | 100% | 99 | 100 | JX567078 | *Bos taurus* | 99% | 99 | | 100 | |  | | |
| 74 | Venison | mince | *Antidorcas marsupialis* | JX567234 | | *Antidorcas marsupialis* | 99% | 99 | 100 | JX567079 | *Ovis aries* | 100% | 79 | | 100 | |  | | |
| 75 | Ostrich | fresh wors | *Struthio camelus* | JX567235 | | *Struthio camelus* | 100% | 100 | 100 | JX567080 | *Struthio camelus* | 100% | 100 | | 100 | |  | | |
| 76 | Gemsbok | biltong | *Oryx gazella* | JX567236 | | *Connochaetes taurinus* | 99% | 66 (SA) | 100 | JX567081 | *Connochaetes taurinus* | 100% | 73 (SA) | | **100 (G)** | |  | | |
| 77 | Kudu | biltong | *Tragelaphus strepsiceros* | JX567237 | | *Tragelaphus oryx* | 98% | 91 | 100 | JX567082 | *Tragelaphus oryx* | 99% | 98 (S) | | 100 (S) | |  | | |
| 78 | Springbok | biltong | *Antidorcas marsupialis* | JX567238 | | *Tragelaphus strepsiceros* | 97% | 92 | 100 | JX567083 | *Tragelaphus strepsiceros* | 98% | 98 | | 100 | |  | | |
| 79 | Ostrich | biltong | *Struthio camelus* | JX567239 | | *Struthio camelus* | 100% | 100 | 100 | JX567084 | *Struthio camelus* | 100% | 100 | | 100 | |  | | |
| 80 | impala | biltong | *Aepyceros melampus* | JX567240 | | *Damaliscus pygargus* | 99% | 88 | 100 | JX567085 | *Damaliscus pygargus* | 100% | 99 | | 100 | |  | | |
| 81 | Kudu | droë wors | *Tragelaphus strepsiceros* | JX567241 | | *Bos taurus* | 99% | 94 | 100 | JX567086 | *Bos taurus* | 99% | 99 | | 100 | |  | | |
| 82 | Ostrich | droë wors | *Struthio camelus* | JX567242 | | *Bos taurus* | 99% | 94 | 100 | JX567087 | *Ovis aries* | 93% | 79 | | 100 | |  | | |
| 83 | Ostrich | biltong | *Struthio camelus* | JX567243 | | *Struthio camelus* | 100% | 100 | 100 | JX567088 | *Struthio camelus* | 100% |  | |  | |  | | |
| 84 | Springbok | biltong | *Antidorcas marsupialis* | JX567244 | | *Aepyceros melampus* | 99% | 99 | 100 | JX567089 | *Aepyceros melampus* | 99% |  | | 100 | |  | | |
| 85 | Beef | biltong | *Bos taurus* | JX567245 | | *Bos taurus* | 100% | 94 | 100 | JX567090 | *Bos taurus* | 99% | 99 | | 100 | |  | | |
| 86 | Beef | biltong | *Bos taurus* | JX567246 | | *Bos taurus* | 100% | 94 | 100 | JX567091 | *Bos taurus* | 100% | 99 | | 100 | |  | | |
| 87 | Kudu | biltong | *Tragelaphus strepsiceros* | JX567247 | | *Oryx gazella* | 100% | 100 | 100 | JX567092 | *Oryx gazella* | 100% | 99 | | 100 | |  | | |
| 88 | Beef | biltong | *Bos taurus* | JX567248 | | *Bos taurus* | 100% | 94 | 100 | JX567093 | *Bos taurus* | 99% | 99 | | 100 | |  | | |
| 89 | doggy | biltong | *Bos taurus* | JX567249 | | *Bos taurus* | 99% | 94 | 100 | JX567094 | *Bos taurus* | 100% | 99 | | 100 | |  | | |
| 90 | Gemsbok | biltong | *Oryx gazella* | JX567250 | | *Bos taurus* | 100% | 94 | 100 | JX567095 | *Bos taurus* | 100% | 99 | | 100 | |  | | |
| 91 | Eland | biltong | *Tragelaphus oryx* | JX567251 | | *Bos taurus* | 100% | 94 | 100 | JX567096 | *Bos taurus* | 99% | 99 | | 100 | |  | | |
| 92 | Kudu | biltong | *Tragelaphus strepsiceros* | JX567252 | | *Bos taurus* | 100% | 94 | 100 | JX567097 | *Bos taurus* | 100% | 99 | | 100 | |  | | |
| 93 | Ostrich | biltong | *Struthio camelus* | JX567253 | | *Macropus robustus* | 99% | 99 | 100 | JX567098 | *Macropus robustus* | 99% | 100 | | 100 | |  | | |
| 93 |  |  |  |  | |  |  |  |  | JX567099 | *Macropus robustus* | 99% | 100 | | U | |  | | |
| 94 | Springbok | biltong | *Antidorcas marsupialis* | JX567254 | | *Bos taurus* | 100% | 94 | 100 | JX567100 | *Bos taurus* | 100% | 99 | | 100 | |  | | |
| 95 | Beef | biltong | *Bos taurus* | JX567255 | | *Bos taurus* | 100% | 94 | 100 | JX567101 | *Bos taurus* | 100% | 99 | | 100 | |  | | |
| 96 | Gemsbok | biltong | *Oryx gazella* | JX567256 | | *Aepyceros melampus* | 99% | 99 | 100 | JX567102 | *Aepyceros melampus* | 99% | 100 | | 100 | |  | | |
| 97 | impala | biltong | *Aepyceros melampus* | JX567257 | | *Aepyceros melampus* | 98% | 99 | 100 | JX567103 | *Aepyceros melampus* | 99% | 100 | | 100 | |  | | |
| 98 | Springbok | biltong | *Antidorcas marsupialis* | JX567258 | | *Equus caballus* | 100% | 96 | 100 | JX567104 | *Equus caballus* | 100% | 97 | | 100 | |  | | |
| 99 | Kudu | biltong | *Tragelaphus strepsiceros* | JX567259 | | *Tragelaphus oryx* | 98% | 91 | 100 | JX567105 | *Tragelaphus oryx* | 99% | 98 (S) | | 100 | |  | | |
| 100 | Game | droë wors | *any game species* | JX567260 | | *Bos taurus* | 100% | 94 | 100 | JX567106 | *Bos taurus* | 100% | 99 | | 100 | |  | | |
| 101 | Eland | biltong | *Tragelaphus oryx* | JX567261 | | *Tragelaphus strepsiceros* | 97% | 92 | 100 | JX567107 | *Tragelaphus strepsiceros* | 98% | 98 | | 100 | |  | | |
| 102 | Springbok | biltong | *Antidorcas marsupialis* | JX567262 | | *Antidorcas marsupialis* | 100% | 99 | 100 | JX567108 | *Antidorcas marsupialis* | 99% | 100 | | 100 | |  | | |
| 103 | Kudu | biltong | *Tragelaphus strepsiceros* | JX567263 | | *Oryx gazella* | 100% | 100 | 100 | JX567109 | *Oryx gazella* | 100% | 99 | | 100 | |  | | |
| 104 | Beef | biltong | *Bos taurus* | JX567264 | | *Bos taurus* | 100% | 94 | 100 | JX567110 | *Bos taurus* | 100% | 99 | | 100 | |  | | |
| 105 | Ostrich | droë wors | *Struthio camelus* | JX567265 | | *Macropus giganteus* | 100% | 93 | 100 | JX567111 | *Macropus robustus* | 89% | 67 (M) | | U | |  | | |
| 105 |  |  |  |  | |  |  |  |  | JX567112 | *Macropus robustus* | 98% | 100(Mr) | | U | |  | | |
| 106 | Springbok | droë wors | *Antidorcas marsupialis* | JX567266 | | *Macropus robustus* | 96% | 96 | U | JX567113 | *Bos taurus* | 100% | 99 | | 100 | |  | | |
| 106 |  |  |  | JX567267 | | *Bos taurus* | 99% | 94 | 100 | JX567114 | *Macropus robustus* | 99% | 100(Mr) | | U | |  | | |
| 107 | Beef | droë wors | *Bos taurus* | JX567268 | | *Bos taurus* | 100% | 94 | 100 | JX567115 | *Bos taurus* | 100% | 99 | | 100 | |  | | |
| 108 | Gemsbok | biltong | *Oryx gazella* | JX567269 | | *Bos taurus* | 99% | 94 | 100 | JX567116 | *Bos taurus* | 100% | 99 | | 100 | |  | | |
| 109 | Springbok | droë wors | *Antidorcas marsupialis* | JX567270 | | *Oryx gazella* | 100% | 100 | 100 | JX567117 | *Oryx gazella* | 100% | 100 | | 100 | |  | | |
| 110 | Kudu | droë wors | *Tragelaphus strepsiceros* | JX567271 | | *Tragelaphus strepsiceros* | 97% | 92 | 100 | JX567118 | *Tragelaphus strepsiceros* | 97% | 98 | | 100 | |  | | |
| 110 |  |  |  | JX567272 | | *Oryx gazella* | 99% | 100 | U |  |  |  |  | |  | |  | | |
| 111 | Beef | droë wors | *Bos taurus* | JX567273 | | *Bos taurus* | 100% | 94 | 100 | JX567119 | *Bos taurus* | 100% | 99 | | 100 | |  | | |
| 112 | Ostrich | droë wors | *Struthio camelus* | JX567274 | | *Bos taurus* | 100% | 94 | 100 | JX567120 | *Ovis aries* | 100% | 79 | | 100 | |  | | |
| 112 |  |  |  |  | |  |  |  |  | JX567121 | *Bos taurus* | 99% | 99 | | 100 | |  | | |
| 113 | Beef | biltong | *Bos taurus* | JX567275 | | *Bos taurus* | 100% | 94 | 100 | JX567122 | *Bos taurus* | 100% | 99 | | 100 | |  | | |
| 114 | Kudu | biltong | *Tragelaphus strepsiceros* | JX567276 | | *Giraffa camelopardalis* | 100% | 100 | 100 | JX567123 | *Giraffa camelopardalis* | 100% | 100 | | 100 | |  | | |
| 115 | Kudu | droë wors | *Tragelaphus strepsiceros* | JX567277 | | *Macropus rufus* | 99% | 97 | 100 | JX567124 | *Bos taurus* | 100% | 99 | | 100 | |  | | |
| 115 |  |  |  | JX567278 | | *Macropus robustus* | 99% | 99 | 100 | JX567125 | *Macropus robustus* | 99% | 100 (Mr) | | U | |  | | |
| 116 | Ostrich | biltong | *Struthio camelus* | JX567279 | | *Bos taurus* | 100% | 94 | 100 | JX567126 | *Bos taurus* | 100% | 99 | | 100 | |  | | |
| 117 | Gemsbok | biltong | *Oryx gazella* | JX567280 | | *Oryx gazella* | 100% | 100 | 100 | JX567127 | *Oryx gazella* | 100% | 99 | | 100 | |  | | |
| 118 | Springbok | biltong | *Antidorcas marsupialis* | JX567281 | | *Antidorcas marsupialis* | 100% | 99 | 100 | JX567128 | *Antidorcas marsupialis* | 99% | 99 | | 100 | |  | | |
| 119 | Springbok | droë wors | *Antidorcas marsupialis* | JX567282 | | *Bos taurus* | 99% | 94 | 100 | JX567129 | *Bos taurus* | 100% | 99 | | 100 | |  | | |
| 120 | Eland | biltong | *Tragelaphus oryx* | JX567283 | | *Oryx gazella* | 100% | 100 | 100 | JX567130 | *Oryx gazella* | 100% | 99 | | 100 | |  | | |
| 121 | Eland | biltong | *Tragelaphus oryx* | JX567284 | | *Connochaetes taurinus* | 100% | 66 (SA) | 100 | JX567131 | *Connochaetes taurinus* | 99% | 73 (SA) | | 100 (G) | |  | | |
| 122 | Kudu | biltong | *Tragelaphus strepsiceros* | JX567285 | | *Oryx gazella* | 100% | 100 | 100 | JX567132 | *Oryx gazella* | 99% | 99 | | 100 | |  | | |
| 123 | Gemsbok | mince | *Oryx gazella* | JX567286 | | *Oryx gazella* | 100% | 100 | 100 | JX567133 | *Oryx gazella* | 100% | 99 | | 100 | |  | | |
| 124 | Venison | mince | *Antidorcas marsupialis* | JX567287 | | *Antidorcas marsupialis* | 100% | 99 | 100 | JX567134 | *Antidorcas marsupialis* | 99% | 99 | | 100 | |  | | |
| 125 | Kudu | droë wors | *Tragelaphus strepsiceros* | JX567288 | | *Bos taurus* | 100% | 94 | 100 | JX567135 | *Ovis aries* | 99% | 79 | | 100 | |  | | |
| 126 | Ostrich | droë wors | *Struthio camelus* | JX567289 | | *Bos taurus* | 99% | 94 | 100 | JX567136 | *Bos taurus* | 99% | 99 | | 100 | |  | | |
| 127 | Game | biltong | *any game species* | JX567290 | | *Bos taurus* | 100% | 94 | 100 | JX567137 | *Bos taurus* | 100% | 99 | | 100 | |  | | |
| 128 | Game | biltong | *any game species* | JX567291 | | *Bos taurus* | 100% | 94 | 100 | JX567138 | *Bos taurus* | 100% | 99 | | 100 | |  | | |
| A01 | Ostrich | biltong | *Struthio camelus* | JX567292 | | *Bos taurus* | 100% | 94 | 100 | JX567139 | *Bos taurus* | 100% | 99 | | 100 | |  | | |
| A02 | Kudu | biltong | *Tragelaphus strepsiceros* | JX567293 | | *Oryx gazella* | 100% | 100 | 100 | JX567140 | *Oryx gazella* | 99% | 99 | | 100 | |  | | |
| A03 | Ostrich | biltong | *Struthio camelus* | JX567294 | | *Struthio camelus* | 99% | 100 | 100 | JX567141 | *Struthio camelus* | 98% | 100 | | 100 | |  | | |
| A04 | Kudu | biltong | *Tragelaphus strepsiceros* | JX567295 | | *Equus burchellii* | 99% | 84 (**) | 100 | JX567142 | *Equus burchellii* | 100% | 98 | | 100 | |  | | |
| A05 | Springbok | biltong | *Antidorcas marsupialis* | JX567296 | | *Connochaetes taurinus* | 99% | 66 (SA) | 100 | JX567143 | *Connochaetes taurinus* | 100% | 73 (SA) | | **100 (G)** | |  | | |
| A06 | Springbok | biltong | *Antidorcas marsupialis* | JX567297 | | *Bos taurus* | 100% | 94 | 100 | JX567144 | *Bos taurus* | 100% | 99 | | 100 | |  | | |
| A07 | Kudu | biltong | *Tragelaphus strepsiceros* | JX567298 | | *Bos taurus* | 100% | 94 | 100 | JX567145 | *Bos taurus* | 97% | 99 | | 100 | |  | | |
| A08 | Ostrich | biltong | *Struthio camelus* | JX567299 | | *Bos taurus* | 100% | 94 | 100 | JX567146 | *Bos taurus* | 100% | 99 | | 100 | |  | | |
| A09 | Ostrich | biltong | *Struthio camelus* | JX567300 | | *Struthio camelus* | 99% | 100 | 100 | JX567147 | *Struthio camelus* | 99% | 100 | | 100 | |  | | |
| A10 | Kudu | biltong | *Tragelaphus strepsiceros* | JX567301 | | *Bos taurus* | 99% | 94 | 100 | JX567148 | *Bos taurus* | 100% | 99 | | 100 | |  | | |
| A11 | Kudu | biltong | *Tragelaphus strepsiceros* | JX567302 | | *Tragelaphus oryx* | 99% | 91 | 100 | JX567149 | *Tragelaphus oryx* | 99% | 98 (S) | | 100 (S) | |  | | |
| A12 | Ostrich | biltong | *Struthio camelus* | JX567303 | | *Connochaetes gnou* | 99% | 72 | 100 | JX567150 | *Connochaetes gnou* | 99% | 55 | | **100 (G)** | |  | | |
| A13 | Springbok | biltong | *Antidorcas marsupialis* | JX567304 | | *Tragelaphus oryx* | 98% | 91 | 100 | JX567151 | *Tragelaphus oryx* | 99% | 98 (S) | | 100 | |  | | |
| A14 | Beef | biltong | *Bos taurus* | JX567305 | | *Bos taurus* | 100% | 94 | 100 | JX567152 | *Bos taurus* | 100% | 99 | | 100 | |  | | |
| A15 | Gemsbok | biltong | *Oryx gazella* | JX567306 | | *Aepyceros melampus* | 99% | 99 | 100 | JX567153 | *Aepyceros melampus* | 99% | 100 | | 100 | |  | | |
| A16 | impala | biltong | *Aepyceros melampus* | JX567307 | | *Bos taurus* | 100% | 94 | 100 | JX567154 | *Bos taurus* | 100% | 99 | | 100 | |  | | |
| 145 | blesbok | fresh | *Damaliscus pygargus*  *phillipsi* | JX567308 | | *Damaliscus pygargus* | 100% | 89 | 100 | JX567155 | *Damaliscus pygargus* | 100% | 99 | | 100 | |  | | |
| 146 | blesbok | fresh | *Damaliscus pygargus*  *phillipsi* | JX567309 | | *Tragelaphus scriptus (SA)* | 99% | 98 | 100 | JX567156 | *Tragelaphus scriptus* | 99% | 93 | | 100 ** | |  | | |
|  |  |  |  |  | |  |  |  |  |  |  |  |  | |  | |  | | |
|  | boots (*) the correct tree topology is not recovered | | |  | |  |  |  |  |  |  |  |  | |  | |  | | |
|  | ML (N): the highest bootstrap support is obtained at subspecies level, from Namibia | | | | | |  |  |  | ML (S) : bootstrap for southern cluster | |  |  | |  | |  | | |
|  | ML boots (SA): indicated for the South African cluster of *Connochaetes taurinus*, higher bootstrap is obtained at the genus level | | | | | | | | | ML (Mr): assignment to a cluster containing *M. robustus* | | | | |  | |  | | |
|  | ML boots (SE) : indicated for *Kobus ellipsiprymnus* cluster, South-East Africa | | | | | |  |  |  | ML (M): bootstrap for cluster with all *Macropus* | | |  | |  | |  | | |
|  | ML boots (**): bootstrap support is provided for the smallest inclusive group as monophyly is not recovered for the species | | | | | | | |  | BLOG (G): 100% assignment to genus | |  |  | |  | |  | | |
|  |  |  |  |  | |  |  |  |  | BLOG(S): 100% assignment to southern cluster | | | |  | |  | |  |  |
|  |  | indicates the better performing method | |  | |  |  |  |  |  |  |  |  | |  | |  | | |
