# Supplementary material for: Where is the game? Wild meat products authentication in South Africa: a case study
Source: Investig Genet. 2013 Mar 1;4:6. doi: 10.1186/2041-2223-4-6 (PMC3621286; doi:10.1186/2041-2223-4-6)

Supplementary Figure 1a.

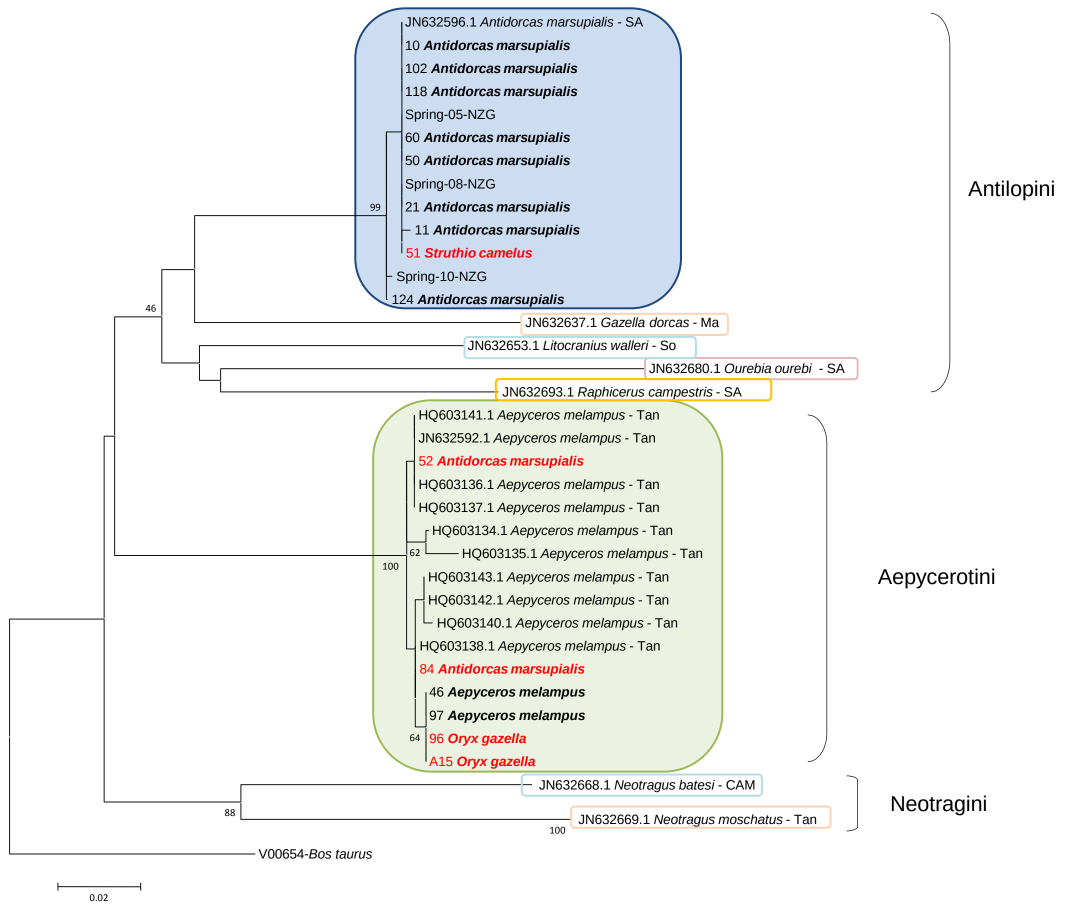

Supplementary Figure 1b.

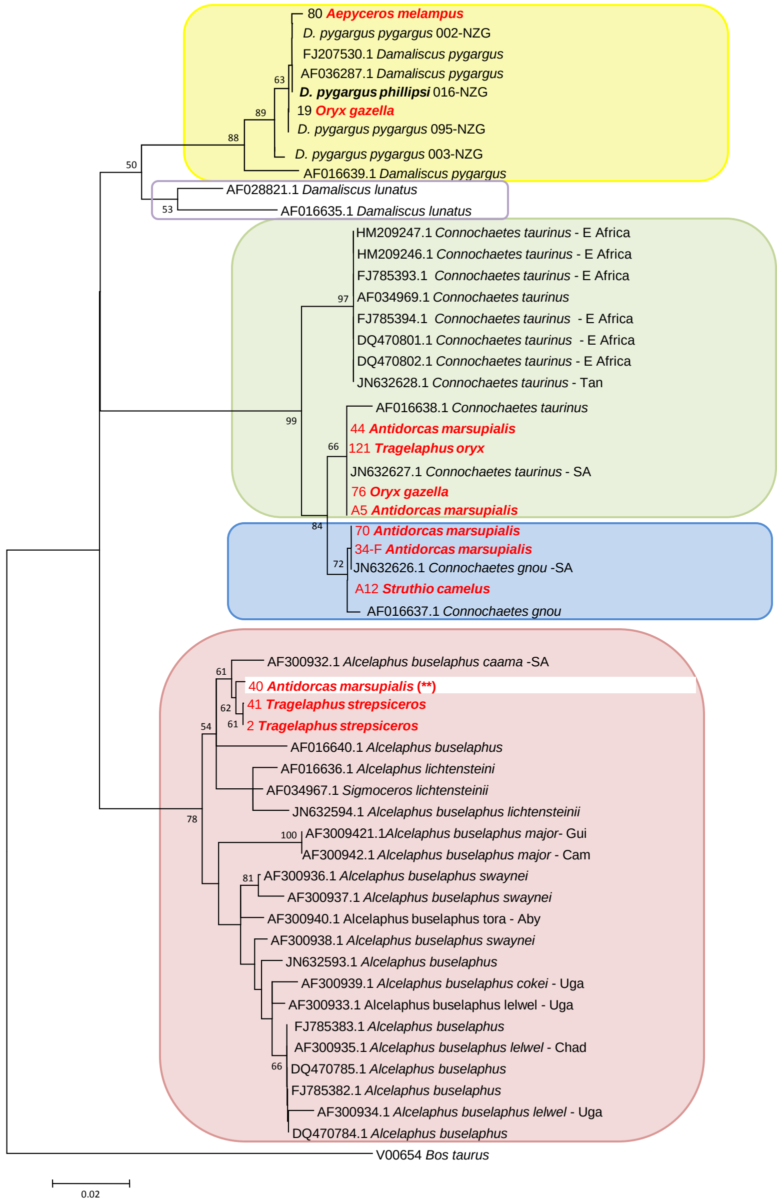

Supplementary Figure 1c.

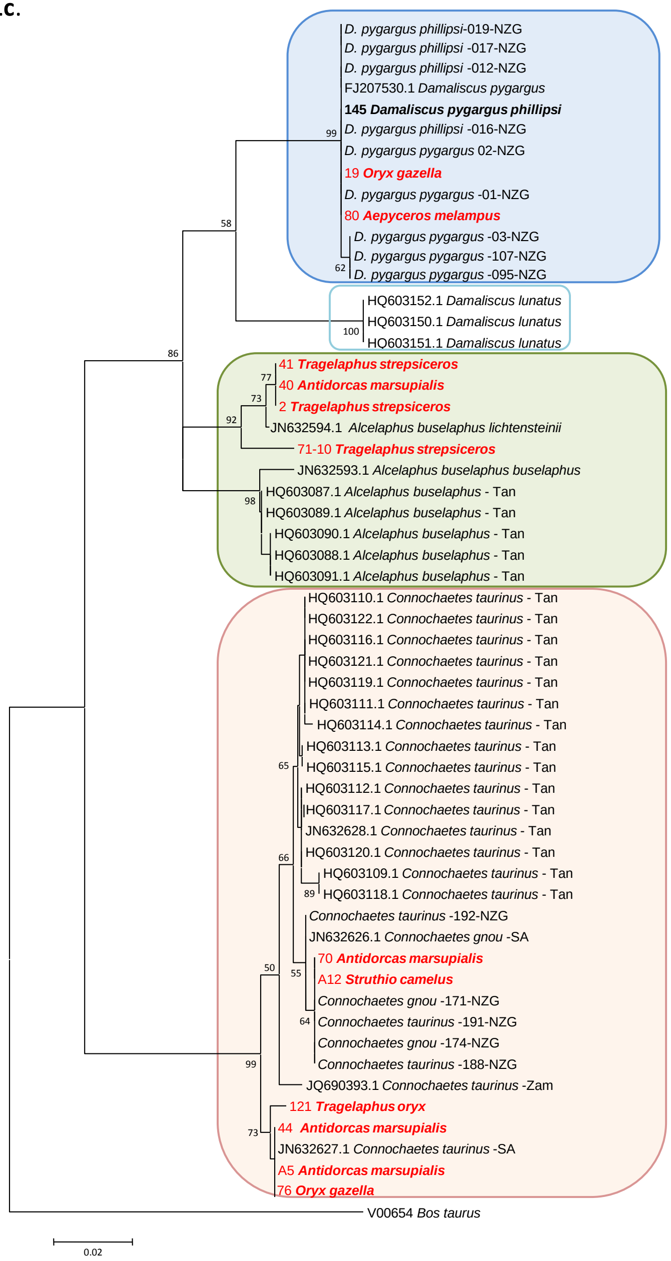

Supplementary Figure 1d.

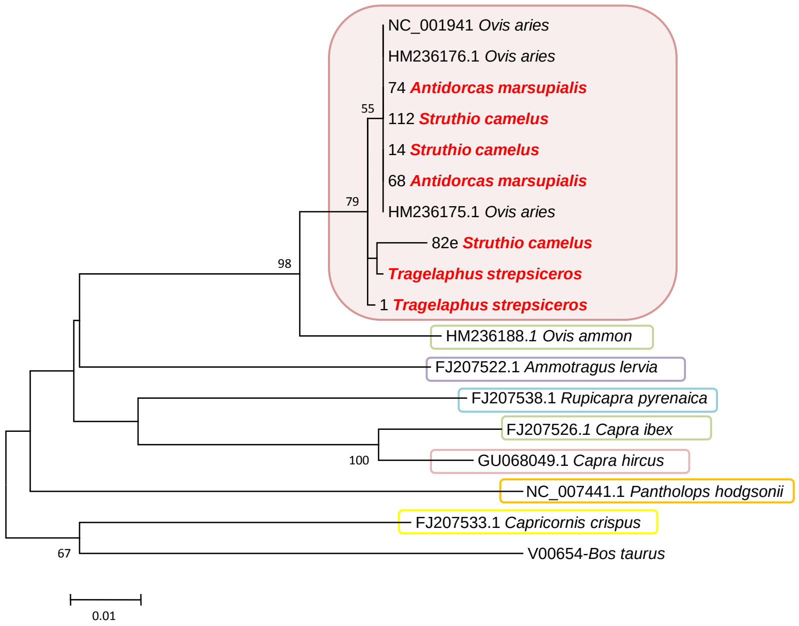

Supplementary Figure 1e.

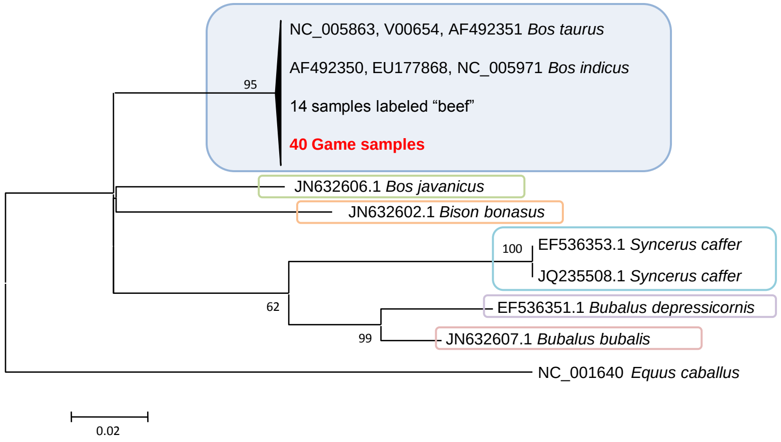

Supplementary Figure 1f.

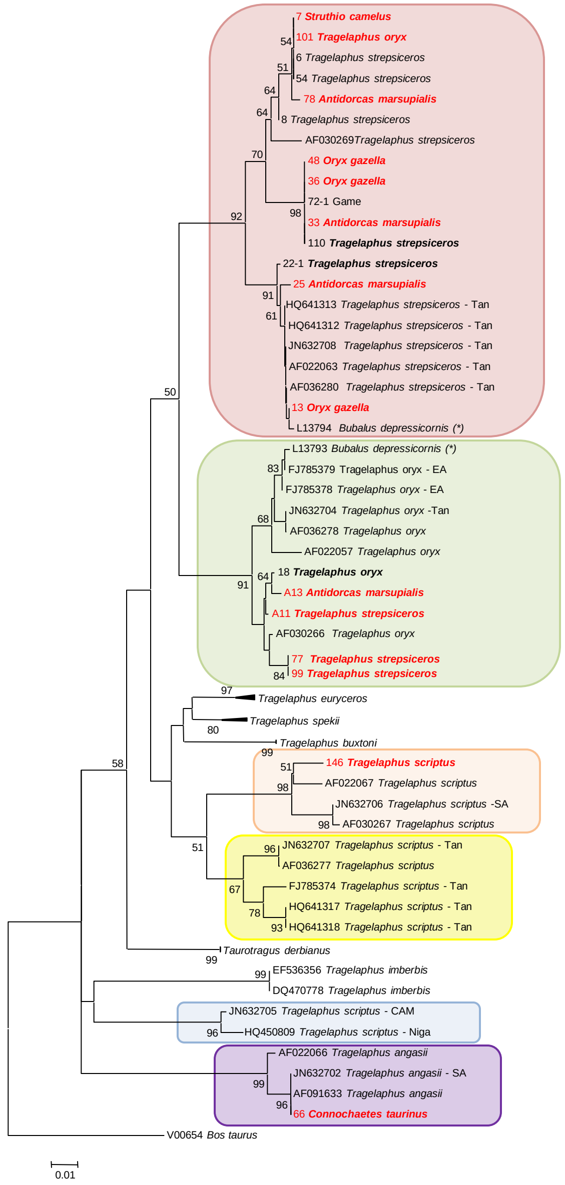

Supplementary Figure 1g.

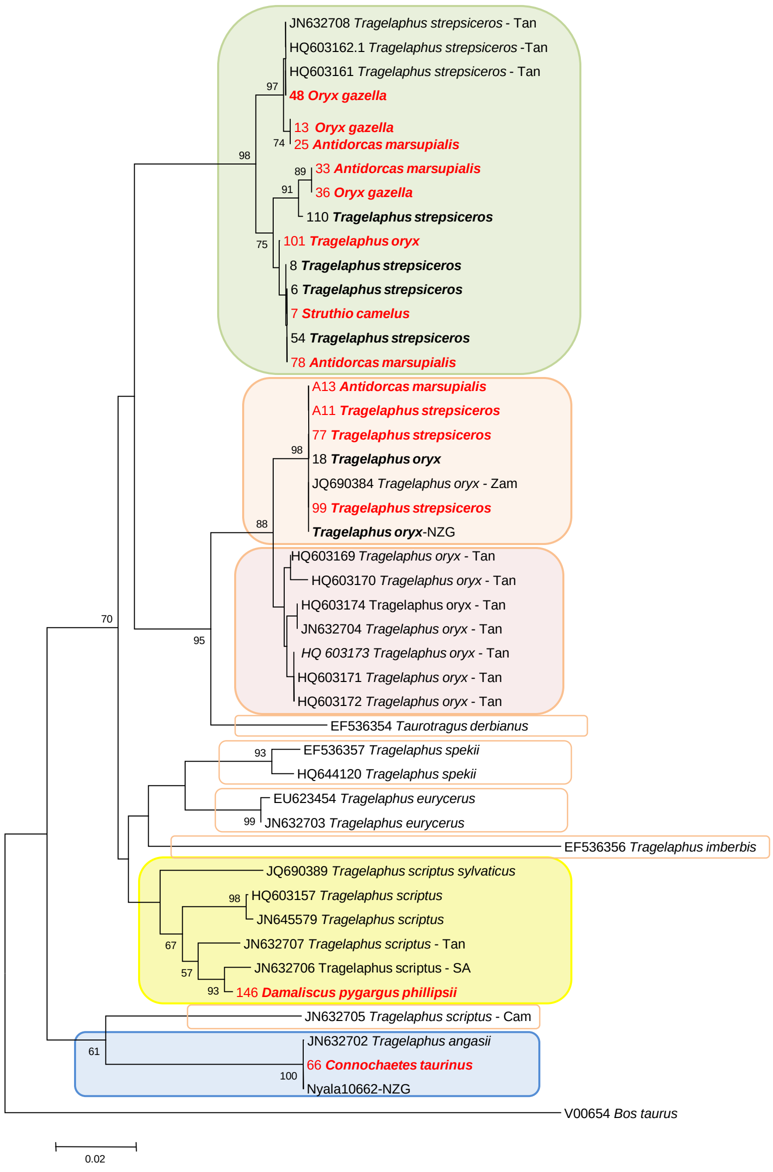

Supplementary Figure 1h.

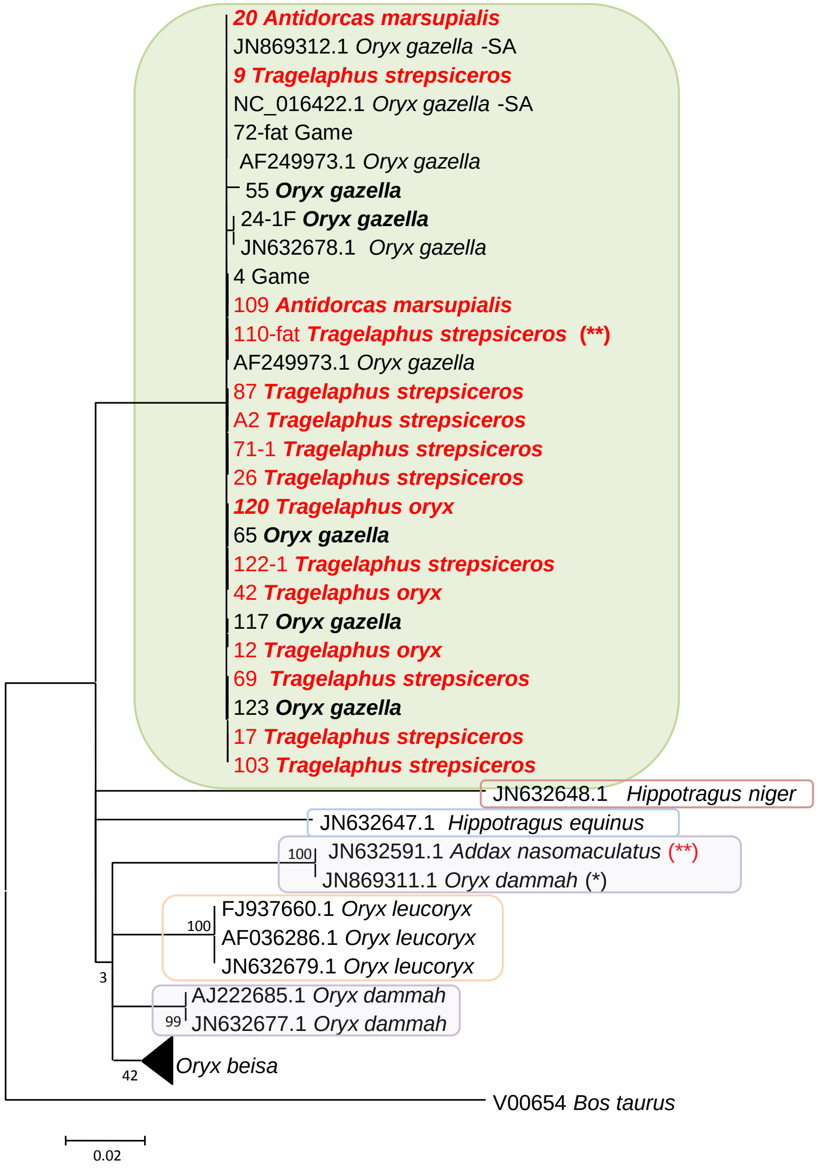

Supplementary Figure 1i.

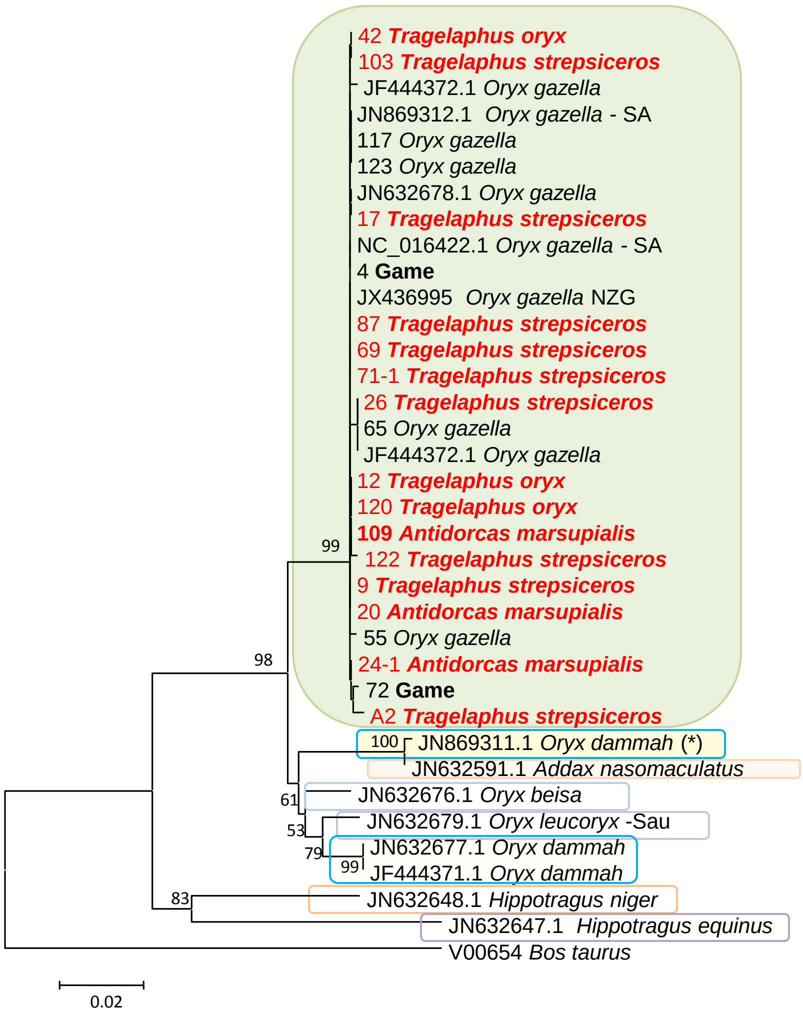

Supplementary Figure 1j.

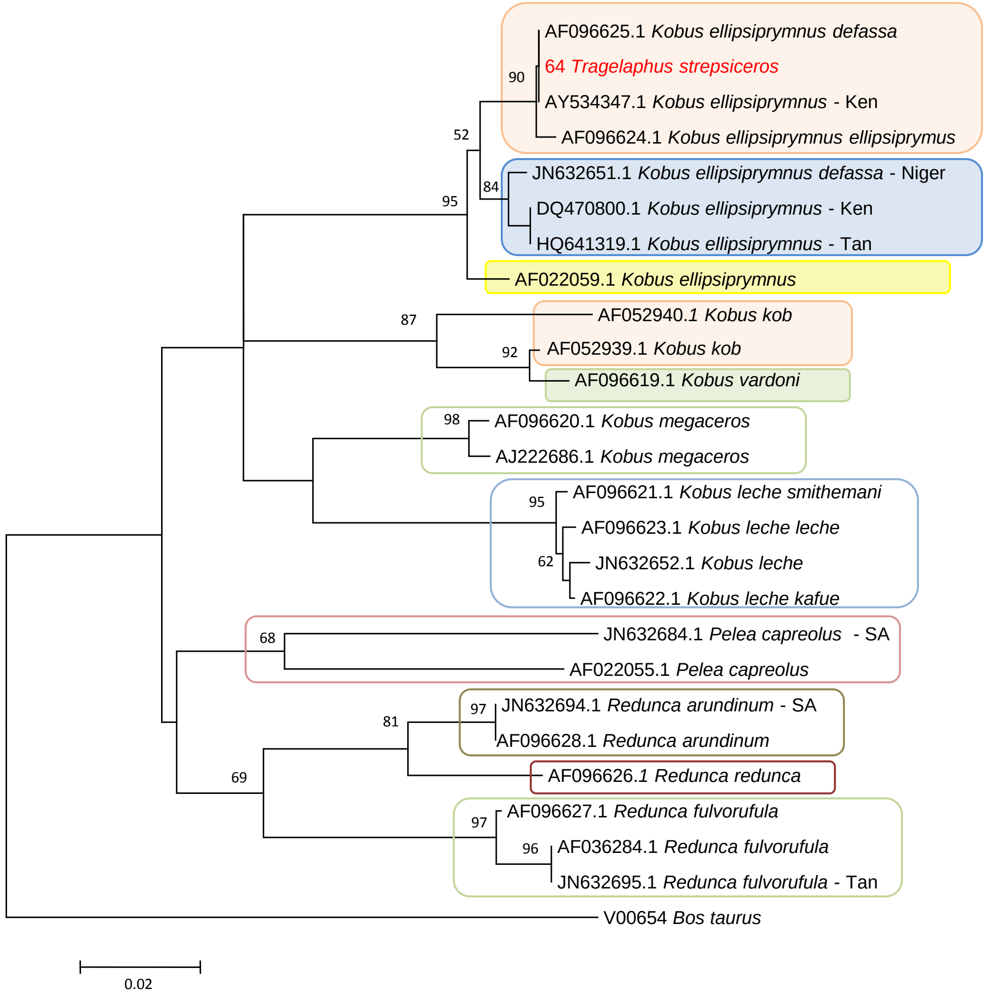

Supplementary Figure 1k.

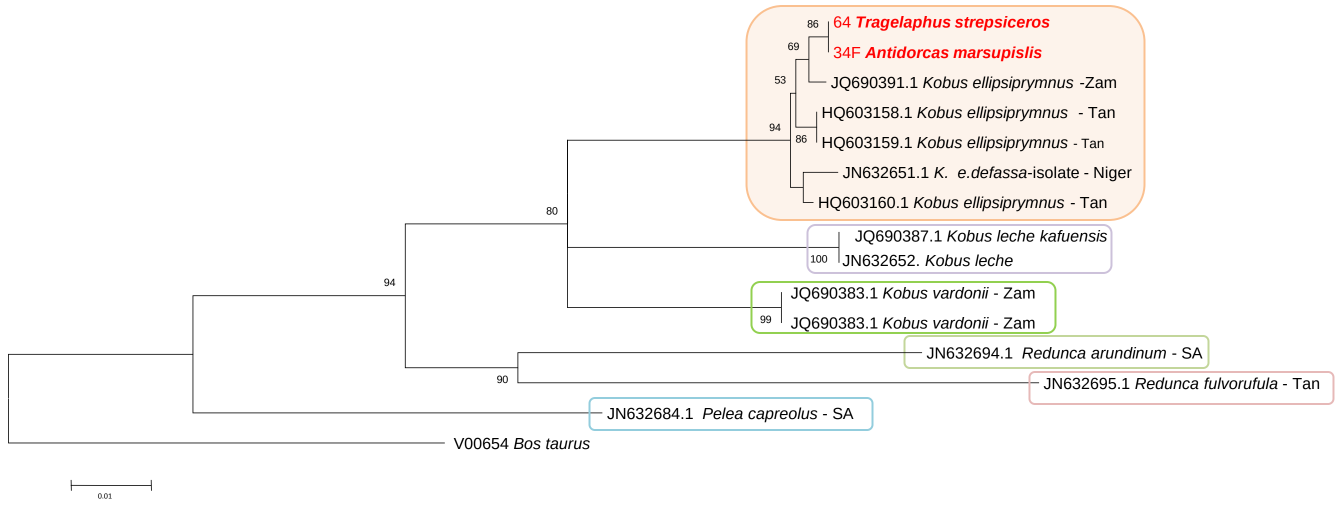

Supplementary Figure 1l.

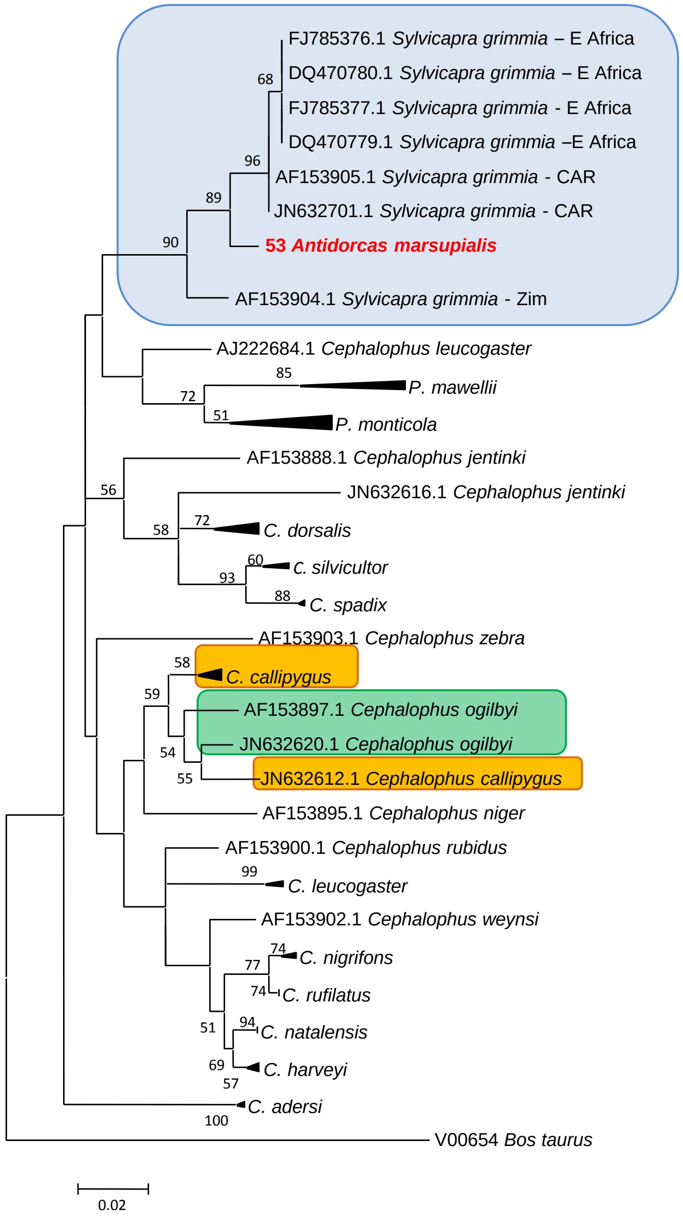

Supplementary Figure 1m.

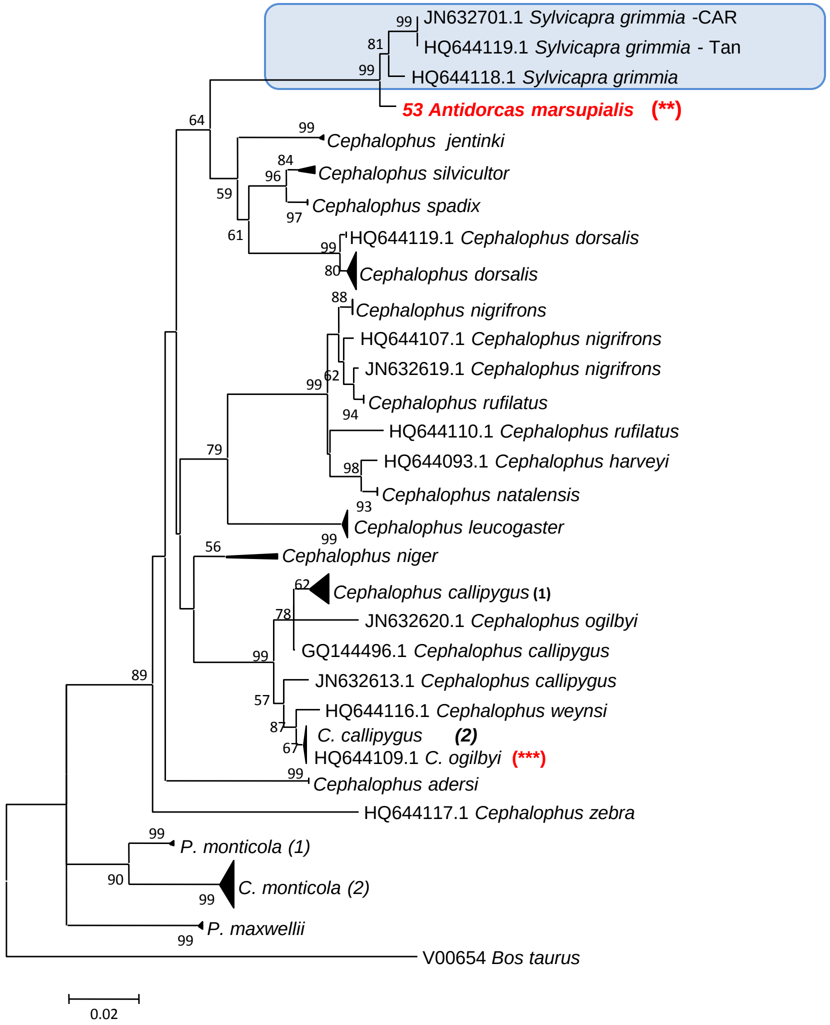

Supplementary Figure 1n.

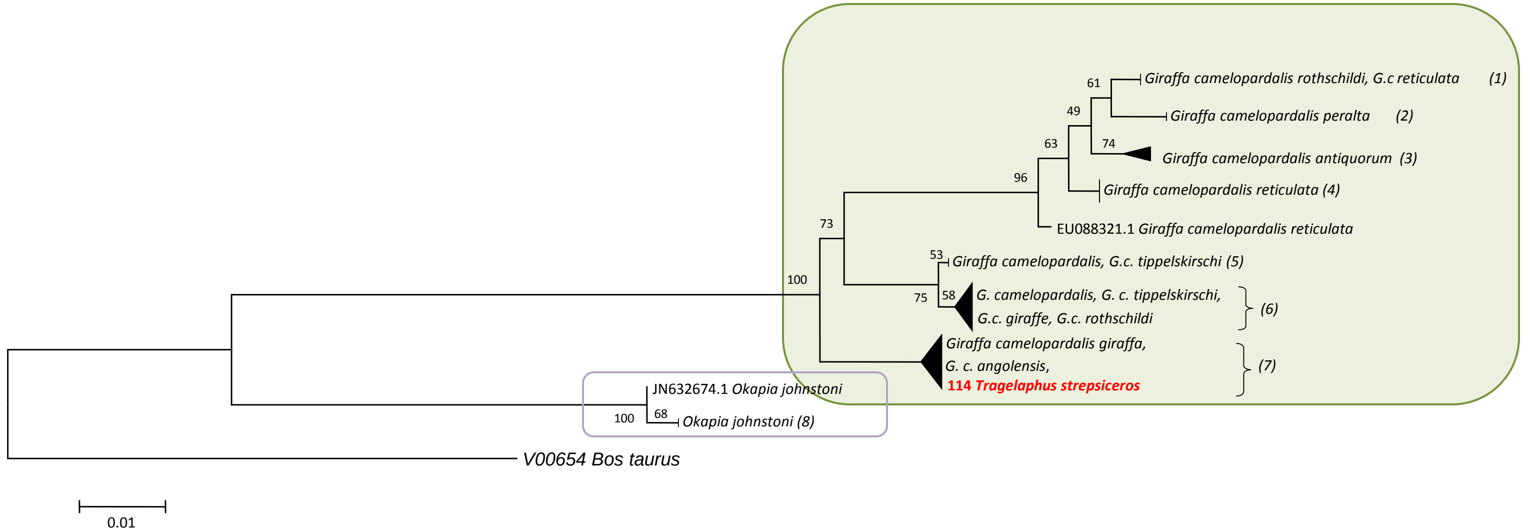

Supplementary Figure 1o.

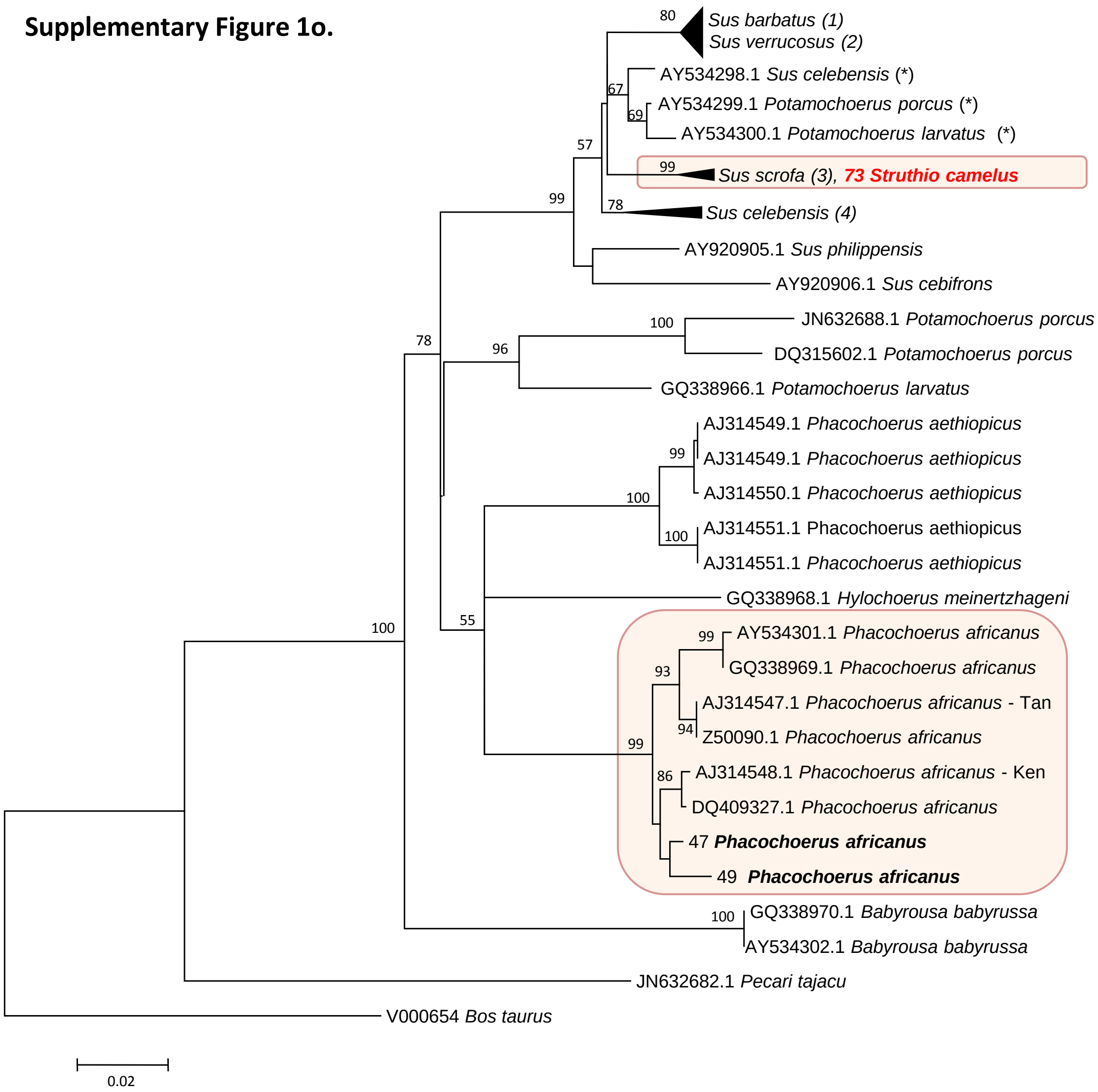

Supplementary Figure 1p.

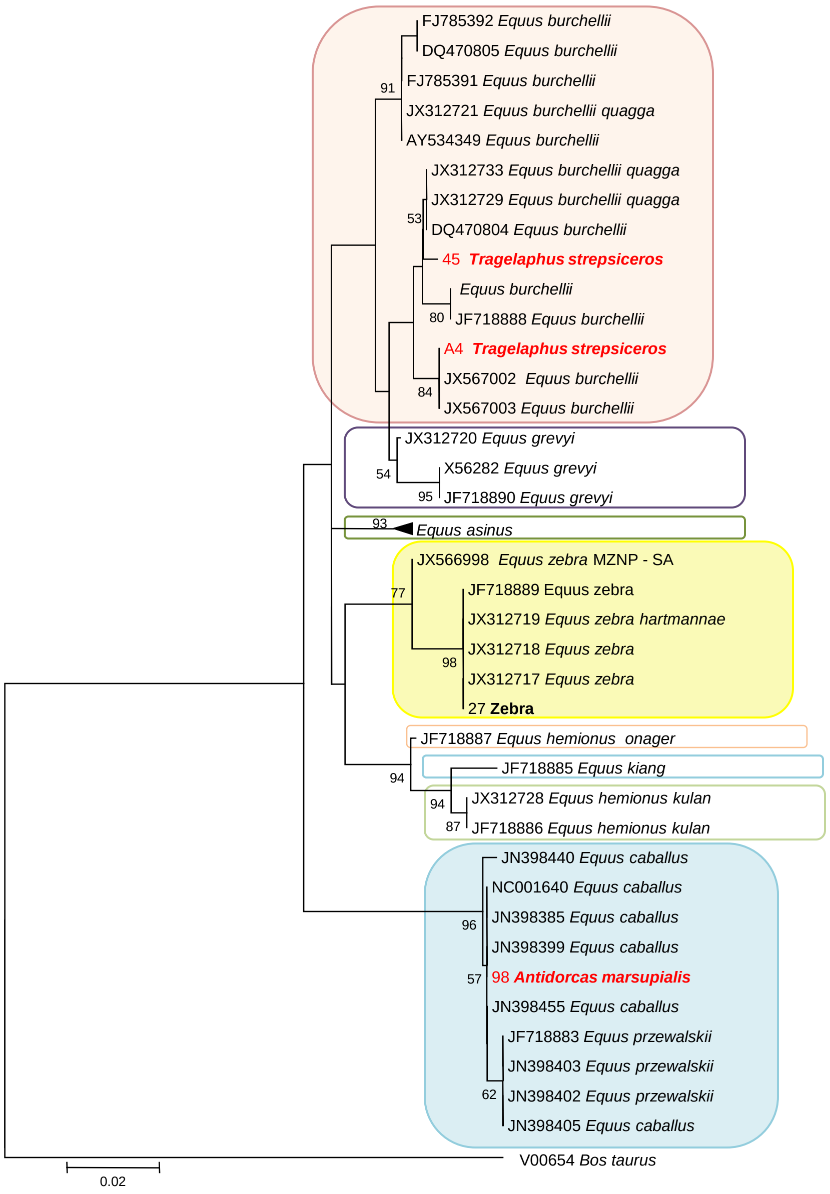

Supplementary Figure 1q.

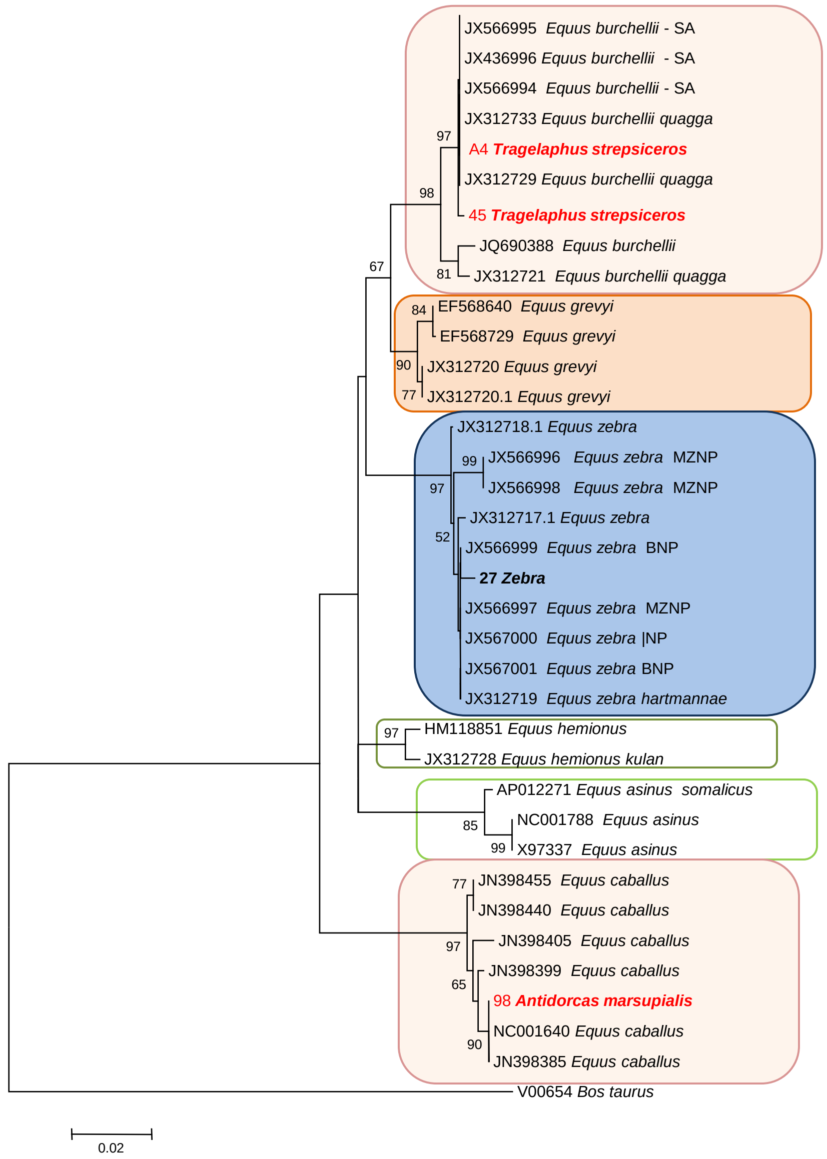

Supplementary Figure 1r.

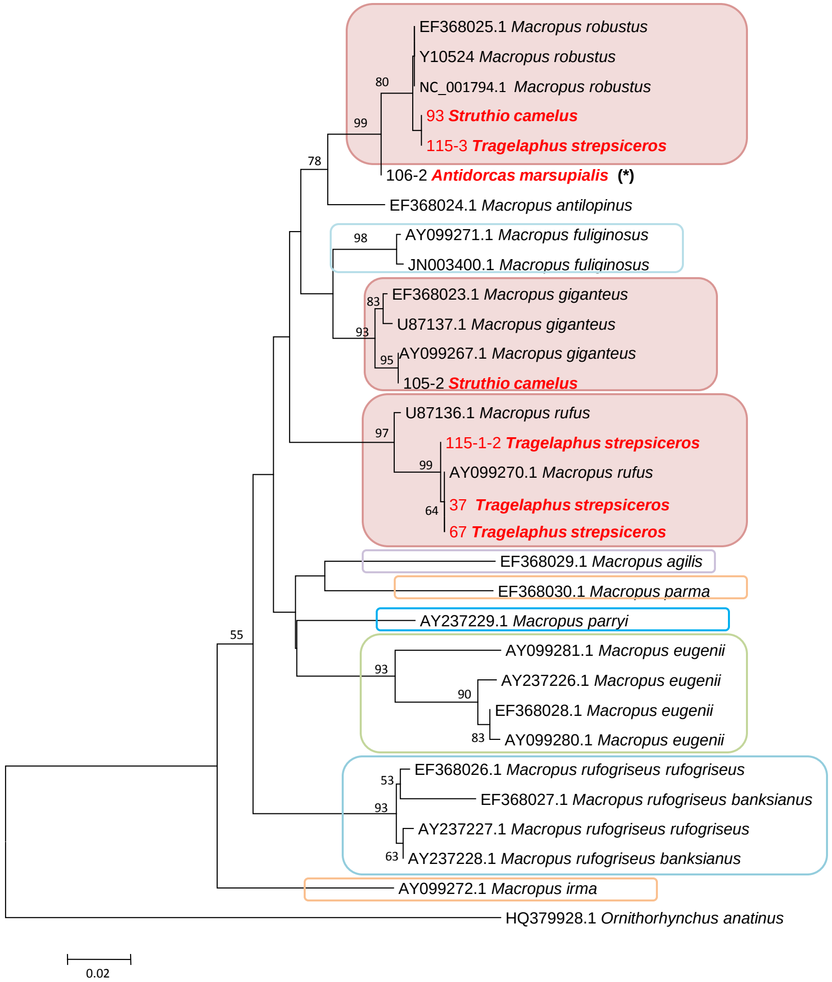

Supplement: Additional file 3 — Consensus Maximum Likelihood trees constructed using Kimura 2-parameter model and 1,000 bootstrap replicates. Branches corresponding to partitions reproduced in less than 50% bootstrap replicates are collapsed; bootstrap support is shown on the nodes. Substitutions are shown in red with the sample ID and species indicated by the commercial label. Species names are kept as the original submissions to GenBank. When known, the specimen geographic origin is indicated: Aby: Abyssinia; BNP: Bontebok National Park; Cam: Cameroon; CAR: Central African Republic; Chad: Chad; E Africa: East Africa; Gui: Guinea; Ken: Kenya; Ma: Mali; MZNP: Mountain Zebra National Park; Niga: Nigeria; Niger: Niger; NZG: National Zoological Gardens; SA: South Africa; Sau: Saudi Arabia; So: Somalia; Tan: Tanzania; Uga: Uganda; Zam: Zambia; Zim: Zimbabwe. Colored blocks indicate clusters defined with 100% assignment efficiency by BLOG. Substitutions are shown in red, food samples ID can be found in Table S2 in Additional file 2. Figure S1a Aepycerotyni and Antilopini COI sequences. ML Tree Log = -2234.00. Figure S1b. Alcelaphinae cytb sequences. ML Tree Log = -1510.45 . ** element not assigned to any class. Figure S1c. Alcelaphini COI sequences. Tree Log L = -1252.20. Figure S1d. Caprini COI sequences. Tree Log L = -2008.41. Figure S1e. Bovini cytb sequences. Tree Log L = -1134.75. Figure S1f. Tragelaphini cytb sequences. * erroneous submission to GenBank. T. eurycerus: JN632703, AF036276, AF022065; T. spekii: EF536357, AJ222680; T. buxtoni: AY667215-216; Tree LogL = -2153.35. BLOG recovered classes with fully assigned (either test or training) elements, which are indicated with colored boxes. Training classes defined for all other species contained 100% assigned elements. Figure S1g. Tragelaphini COI sequences. Tree LogL = -2146.42. BLOG recovered classes with fully assigned elements, which are indicated with colored boxes. Uncolored boxes: other defined training classes with correct classified el [file 2041-2223-4-6-S3.pdf]
